# Associations between dietary patterns and the incidence of total and fatal cardiovascular disease and all-cause mortality in 116,806 individuals from the UK Biobank: a prospective cohort study

**DOI:** 10.1186/s12916-021-01958-x

**Published:** 2021-04-22

**Authors:** Min Gao, Susan A. Jebb, Paul Aveyard, Gina L. Ambrosini, Aurora Perez-Cornago, Jennifer Carter, Xinying Sun, Carmen Piernas

**Affiliations:** 1 School of Public Health, Peking University Health Science Centre, Beijing, China; 2 Nuffield Department of Primary Care Health Sciences, University of Oxford, Radcliffe Primary Care Building, Radcliffe Observatory Quarter, Woodstock Road, Oxford, OX2 6GG UK; 3 NIHR Oxford Biomedical Research Centre, University of Oxford, Oxford, UK; 4 School of Population and Global Health, University of Western Australia, 35 Stirling Highway, Crawley, Perth, Western Australia 6009 Australia; 5 Nuffield Department of Population Health, University of Oxford, Oxford, UK; 6 Cancer Epidemiology Unit, Nuffield Department of Population Health, University of Oxford, Oxford, UK

**Keywords:** Dietary pattern, Reduced rank regression, Cardiovascular disease, All-cause mortality

## Abstract

**Background:**

Traditionally, studies investigating diet and health associations have focused on single nutrients. However, key nutrients co-exist in many common foods, and studies focusing solely on individual nutrients may obscure their combined effects on cardiovascular disease (CVD) and all-cause mortality. We aimed to identify food-based dietary patterns which operate through excess energy intake and explain high variability in energy density, free sugars, saturated fat, and fiber intakes and to investigate their association with total and fatal CVD and all-cause mortality.

**Methods:**

Detailed dietary data was collected using a 24-h online dietary assessment on two or more occasions (*n* = 116,806). We used reduced rank regression to derive dietary patterns explaining the maximum variance. Multivariable Cox-proportional hazards models were used to investigate prospective associations with all-cause mortality and fatal and non-fatal CVD.

**Results:**

Over an average of 4.9 years of follow-up, 4245 cases of total CVD, 838 cases of fatal CVD, and 3629 cases of all-cause mortality occurred. Two dietary patterns were retained that jointly explained 63% of variation in energy density, free sugars, saturated fat, and fiber intakes in total. The main dietary pattern was characterized by high intakes of chocolate and confectionery, butter and low-fiber bread, and low intakes of fresh fruit and vegetables. There was a positive linear association between the dietary pattern and total CVD [hazard ratio (HR) per *z-*score 1.07, 95% confidence interval (CI) 1.04–1.09; HR_total CVD_ 1.40, 95% CI 1.31–1.50, and HR_all-cause mortality_ 1.37, 95% CI 1.27–1.47 in highest quintile]. A second dietary pattern was characterized by a higher intakes of sugar-sweetened beverages, fruit juice, and table sugar/preserves. There was a non-linear association with total CVD risk and all-cause mortality, with increased risk in the highest quintile [HR_total CVD_ 1.14, 95% CI 1.07–1.22; HR_all-cause mortality_ 1.11, 95% CI 1.03–1.19].

**Conclusions:**

We identified dietary patterns which are associated with increased risk of CVD and all-cause mortality. These results help identify specific foods and beverages which are major contributors to unhealthy dietary patterns and provide evidence to underpin food-based dietary advice to reduce health risks.

**Supplementary Information:**

The online version contains supplementary material available at 10.1186/s12916-021-01958-x.

## Background

Reducing the burden of cardiovascular disease (CVD) is a top public health priority in the UK and worldwide [1]. A poor diet is a major contributor to morbidity and premature mortality, especially CVD [2, 3], in part by promoting excess weight, but also by raising total cholesterol and low-density lipoproteins concentrations (LDL) and increasing the risk of diabetes and hypertension [3]. Traditionally, the vast majority of epidemiological studies investigating diet and health associations have usually focused on single nutrients and this evidence is reflected in current dietary recommendations [4–6]. These emphasize the importance of achieving and maintaining a healthy weight, reductions in saturated fatty acids (SFAs) and free sugars [7, 8], and increases in dietary fiber [5]. High dietary energy density and free sugars are associated with increased risk of weight gain [8] which can further increase CVD and mortality risk [8, 9], while SFAs increase total blood cholesterol and LDL [10, 11]. However, other recent meta-analyses and observational studies have not found evidence for a beneficial effect of reducing SFA intake on CVD and total mortality [12, 13], or found protective effects against stroke [14]. Dietary fiber may lower the risk of CVD, through improved glucose control and lower serum cholesterol concentration [15].

However, despite years of public health efforts, population dietary change has been slow [1, 16]. This may reflect in part the difficulties of translating present dietary recommendations into food-based public health advice [17], and some existing recommendations are not universally echoed across countries [18]. The public have frequently been confused by apparently conflicting messages, for example about the importance of reducing saturated fat or free sugars [12], without recognizing that these nutrients frequently co-exist in foods and that the consequence may be a diet that is high in both saturated fats and free sugars and they may have synergistic effects on health. Dietary guidelines which focus on foods rather than individual nutrient recommendations could help avoid confusion and avoid inadvertent increases in one nutrient of concern at the expense of another. Despite the inclusion of some food-based recommendations in recent dietary guidelines (especially regarding fruits, vegetables, dairy), nutrient-based advice still remains the most common, often co-existing with food-based guidance, as seen in the latest release of the Dietary Guidelines for Americans 2020–2025 [6].

Increasingly, researchers have sought to characterize complex dietary patterns using either a priori (based on adherence to a specific patterns, e.g., Mediterranean diet, or a score which reflects overall dietary quality) [19] or a posteriori (based on the observed dietary intake using empirical methods such as factor analysis or principal component analysis (PCA)) [20, 21]. Reduced rank regression (RRR) is a data-dimension reduction technique that aims to identify the combination of food groups that explain the maximum amount of variation in a set of response variables (e.g., nutrients) hypothesized to be on the causal pathway between diet and health outcomes. This approach can test a priori hypotheses of the pathophysiology of disease [22]. To our knowledge, only six longitudinal cohort studies have examined overall CVD risk and/or all-cause mortality using RRR, but all included smaller populations and none was focused in the UK (Additional file 1: Table S1). This population-specificity is important given that dietary patterns can vary substantially even when nutrient intakes are broadly similar, owing to cultural differences in food preference.

Using data from the UK Biobank study, we aimed to identify food-based dietary patterns explaining the variability in known dietary risk factors which operate through excess energy intake, such as energy density, free sugars, saturated fat, and low fiber intakes, and to investigate their association with total and fatal cardiovascular disease (CVD) and all-cause mortality.

## Methods

### Study design and participants

UK Biobank is a prospective study that recruited 502,536 participants aged 37 to 73 at baseline (between 2006 and 2010) with participants’ data linked to hospital and mortality records [23]. Participants completed a full baseline assessment, including self-reported measurements via touch-screen questionnaires as well as a verbal interview collecting a wide range of information on socio-demographic factors, lifestyle and behavioral factors, and a medical history. Physical measurements (i.e., height, weight), blood and urine samples were also taken. UK Biobank protocols and study details can be found elsewhere (http://www.ukbiobank.ac.uk/wp-content/uploads/2011/11/UK-Biobank-Protocol.pdf). The UK Biobank study was conducted according to the Declaration of Helsinki and ethical approval was granted by the North West Multi-Centre Research Ethics Committee (reference number 06/MRE08/65). At recruitment, all participants gave informed consent to participate and be followed-up through data-linkage.

### Study measures

#### Dietary assessment

All UK Biobank participants who had provided an email address at recruitment were invited to complete the 24-h online dietary assessment (Oxford WebQ), which is a web-based 24-h dietary assessment tool developed and evaluated for use in large population studies [24]. The Oxford WebQ was collected at baseline and on up to four separate occasions (cycle 1: February 2011 to April 2011; cycle 2: June 2011 to September 2011; cycle 3: October 2011 to December 2011; cycle 4: April 2012 to June 2012) [25]. Recorded food and drinks were classified into 50 groups according to their nutrient profile or culinary use and closely following the classification used in the UK National Diet and Nutrition Survey and (Additional file 1: Table S2). From the original sample of UK Biobank participants, we used data from participants that completed a dietary assessment on two or more occasions in order to better reflect usual intakes. The Oxford WebQ automatically generated total nutrient intakes as well as intakes from each food/beverage collected at each assessment. We calculated average nutrient and food group intakes across all dietary assessments after removing participants with implausible energy intakes [26]. The Oxford WebQ has been validated against biomarkers [27] and compared to interviewer-administered 24 h recalls [28] and showed acceptable reproducibility when using at least 2 dietary assessments [29].

#### Outcome ascertainment

Outcomes were defined as primary or secondary events using hospital admission and death registry data linked to the UK Biobank. Total CVD was defined as a hospital admission or death using ICD-10 which included coronary heart disease (CHD; I20–I25), congestive heart failure or cardiomyopathy (CHF; I50, I50.1, 150.9, I11.0, I13.0, I13.2, I42, I43.1), and total stroke (I60–I64). Fatal CVD events were measured by I00–I25, I27–I88, and I95–I99. The hospital registry-based follow-up ended on 31st March 2017, in England; 31st October 2016, in Scotland; and 29th February 2016, in Wales. Individuals were censored on these dates, the time of event in question, or the time of death, whichever occurred first. Death registry included all deaths that occurred before 30th April 2020 in England, Wales, and Scotland.

### Statistical analyses

#### Identification of dietary patterns

The RRR analysis was used to identify dietary patterns that could explain the maximum variation in a set of nutrient response variables hypothesized to be on the causal pathway between predictors (food groups) and outcomes (CVD and all-cause mortality events). We selected the following response variables which contribute to excess energy intake: energy density (kJ/g), SFA (% total energy), free sugars (% total energy), and fiber density (g/MJ) as there is evidence for their role in the development of CVD and mortality [7, 8, 10, 15]. Energy density (kJ/g) is a proxy for high energy intake and was calculated as the amount of energy (kJ) divided by the total food weight (g) excluding beverages because of their disproportionate influence on the total volume (g) of the diet [30]. Fiber density (g/MJ) was calculated by dividing total dietary fiber intake (g) by total daily energy intake (kJ) then multiplying by 1000.

Respondents were assigned a *z-*score for each dietary pattern which quantified how much their reported dietary intake reflected each dietary pattern relative to other respondents in the study sample. The RRR model calculates dietary pattern *z*-scores for each respondent as a linear, weighted combination of all of their standardized food group intakes by using weights unique to each dietary pattern. An increasing intake of food groups having a positive factor loadings increases the dietary pattern *z*-score, while an increasing intake of food groups with negative factor loadings decreases the dietary pattern *z*-score. The number of extracted patterns depends on the number of response variables, and dietary patterns which explained individually more than 20% of variation in response variables were retained. To test the robustness of the derived dietary patterns, a random sample cross-validation procedure was performed in which 50 random subsets were used as test sets [31]. The association between the retained dietary patterns and response variables was assessed by correlation coefficients. Distributions of outcomes, demographic, socioeconomic status, behavioral risk factors, medical conditions, and dietary intake were compared to examine the differences across quintiles of dietary pattern scores (chi-squared test for categorical variables and ANOVA tests for continuous variables).

#### Associations between dietary patterns with outcomes

Multivariable Cox proportional hazards models with age as the underlying timescale variable were used to estimate HRs (hazard ratio) for total (fatal and non-fatal combined), fatal CVD risk, and all-cause mortality with 95% CIs (confidence interval) for each increment in the *z*-score of the dietary patterns as well as for quintiles, using the floated absolute risk method [32]. A quadratic term for dietary pattern *z*-scores was included in the model where we found evidence of non-linearity. The proportional hazards assumption was based on Schoenfeld residuals and was not violated for the variables of interest in the adjusted model (*P* > 0.05). Person-time of follow-up was calculated from the age at which the last dietary assessment was completed until the age at which the event happened (CVD or death) or the end of censoring (March 2017), whichever came first. Trend tests were performed by including the median score of each pattern quintile as a continuous variable in the models, the lowest quintile was used as the reference. Restricted cubic splines were computed with five knots to visually explore non-linear associations between dietary patterns and outcomes. All analyses were stratified by sex (men, women) and region (Scotland, Wales, England) and adjusted for ethnicity (others white), Townsend index of deprivation (a composite measure of deprivation based on unemployment, non-car ownership, non-home ownership, and household overcrowding, categorized as quintiles 1–5, with high index indicating more deprivation), education group (higher degree, any school degree, vocational qualifications, other), smoking status (never, current, previous), physical activity (low, moderate, high), energy intake (log transformed), and menopause status (N/A, no, yes). For details on the derivation of these covariates, see Additional file 1: Table S3.

#### Sensitivity, exploratory, and heterogeneity analyses

Sensitivity analyses were conducted to exclude participants who had a CVD event within 2 years after completing their last 24-h online dietary assessment to account for reverse causality (*N* = 115,532). Additionally, the RRR analysis was repeated among participants providing 3+ (*N* = 72,912), 4+ (*N* = 33,760), or 5+ (*N* = 5403) 24-h online dietary assessments to test whether the derived dietary patterns might be influenced by the number of days of dietary reporting.

To examine the potential roles of adiposity measures and blood biomarkers in these relationships, we used multivariable linear regression to calculate geometric mean concentrations of body mass index (BMI) and biomarkers of CVD risk by quintiles of dietary patterns for baseline measurements of systolic blood pressure (SBP), DBP (diastolic blood pressure), HbA1c (glycated hemoglobin A1c), LDL, and HDL (high-density lipoprotein). This cross-sectional analysis included 26,277 participants with BMI and blood biomarkers measured at baseline, in addition to at least 2 dietary assessments (the first one at baseline and the subsequent ones during the follow-up).

Additionally, BMI categories, diagnosed hypertension, diabetes, or high cholesterol were assessed as potential mediators using likelihood ratio tests. Each of these variables was added one at a time to the main model described above, and an additional model was also fitted including all potential mediators.

Heterogeneity in the associations between dietary patterns and risk of total CVD was assessed for sex, age at recruitment (< 60 years or ≥ 60 years), smoking status, BMI group, and presence of any of the risk factors (hypertension, diabetes, and high cholesterol), using likelihood ratio test.

SAS (version 9.4; SAS Institute) was used to conduct RRR. Descriptive statistics and regressions were conducted using Stata (version 14; StataCorp LP). A two-sided *p* value of < 0.05 was defined as statistically significant.

## Results

A total of 116,806 participants were included in all analyses after exclusions. Participants were excluded for the following reasons: did not provide any dietary data (*n* = 291,514), only provided only one dietary questionnaire (*n* = 84,166), CVD occurred before baseline assessment (*n* = 6422), CVD occurred before the last dietary questionnaire (*n* = 1337), pregnancy (*n* = 108), missing data for the key confounders (*n* = 702), response variables (*n* = 10), medical conditions (*n* = 372), and implausible energy intake (over reporters: *n* = 119, under reporters: *n* = 980). (Additional file 1: Figure S1).

**Fig. 1:**
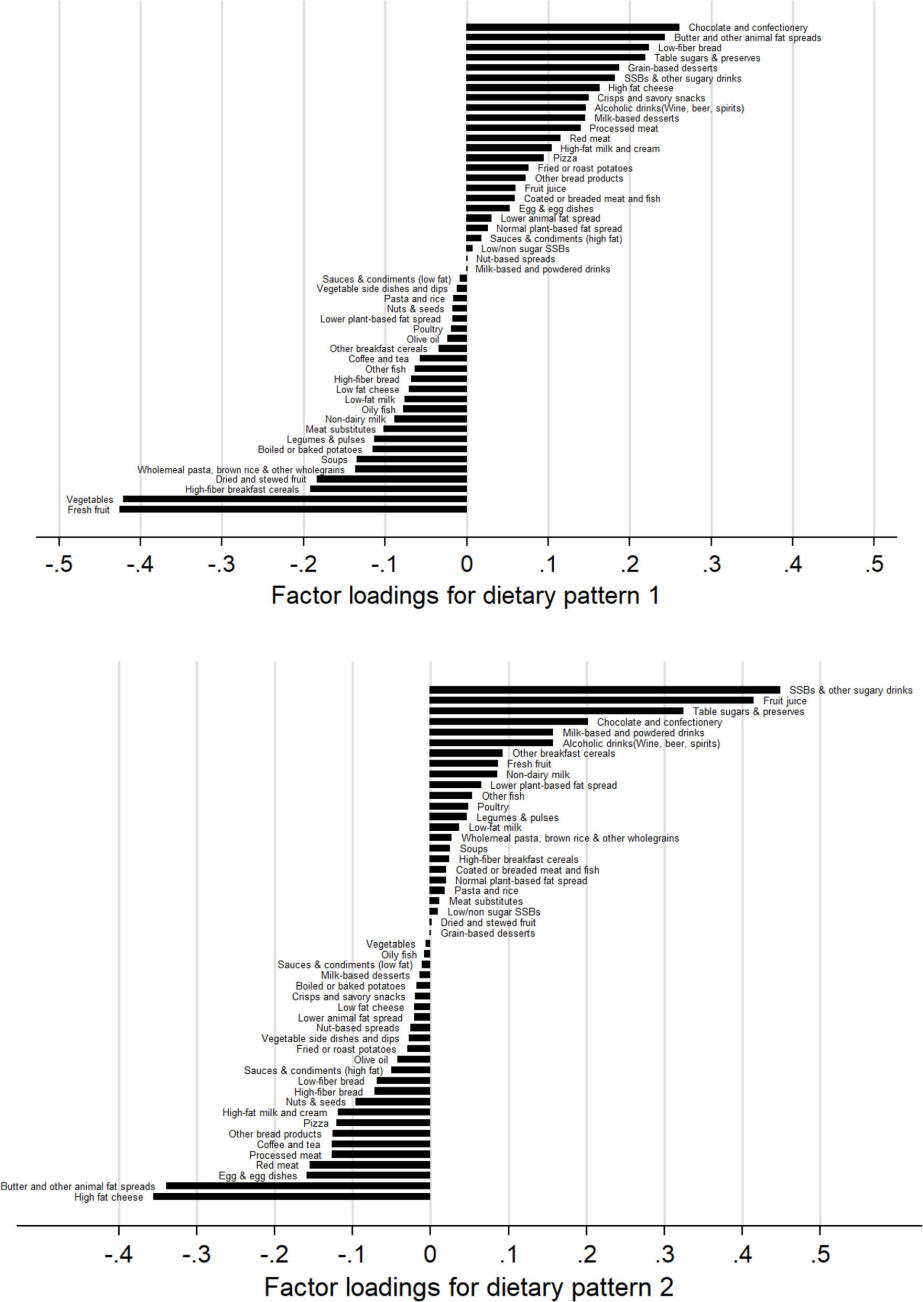
Factor loadings for food groups in each dietary pattern. Note: %E, proportion of total energy intake

The RRR analysis identified two major dietary patterns that could consistently explain the greatest amount of shared variation in all response variables (43% for dietary pattern 1, 20% for dietary pattern 2) (Additional file 1: Table S4). Dietary pattern 1 was characterized by positive loadings for chocolate and confectionery, butter and other animal fat spreads (primarily butter), and low-fiber bread and was strongly negatively associated with fresh fruit, vegetables, and high-fiber breakfast cereals. Dietary pattern 2 was characterized by positive loadings for sugar-sweetened beverages (SSBs), fruit juice, and table sugar and preserves and negative loadings for high fat cheese and butter (Fig. 1). Sensitivity analyses showed that the derived dietary patterns from RRR analyses were consistent regardless of the number of 24 h dietary assessments provided (Additional file 1: Figures S2-S5).

A higher proportion of men, of younger age, with higher Townsend index, current smokers, less physical activity, higher prevalence of obesity, or hypertension were found across higher quintiles of dietary pattern 1 (Table 1, Additional file 1: Table S5). A lower proportion of current smokers, with less physical activity, and lower prevalence of obesity, hypertension, diabetes, and high cholesterol were found across higher quintiles of dietary pattern 2.

**Table 1:** Baseline characteristics of participants in two main dietary patterns (*N* = 116,806)

	Total (*N* = 116,806)	Dietary pattern 1				Dietary pattern 2			
		Quintile 1 (*N* = 23,362)	Quintile 3 (*N* = 23,361)	Quintile 5 (*N* = 23,361)	*P* value*	Quintile 1 (*N* = 23,362)	Quintile 3 (*N* = 23,361)	Quintile 5 (*N* = 23,361)	*P* value*
**Demographics**									
Men (%)	42.7	28.7	40.1	63.4	<0.001	43.7	38.4	51.1	<0.001
Age (years)^†^	55.9 ± 7.8	57.2 ± 7.3	56.1 ± 7.7	53.9 ± 8.1	<0.001	55.9 ± 7.7	56.1 ± 7.8	55.1 ± 8.1	<0.001
White (%)	96.7	96.5	96.8	96.6	0.212	97.6	97.2	94.6	<0.001
**Socioeconomic status**									
Townsend index (quintile 5)	19.9	19.3	18.6	22.5	<0.001	20.8	18.8	21.4	<0.001
Higher degree group (college, university or professional degree/qualification) (%)	52.1	56.9	52.7	44.9	<0.001	53.5	52.0	50.6	<0.001
**Behavioral risk factors**									
Current smoker (%)	6.8	3.8	5.5	12.6	<0.001	8.5	6.0	7.1	<0.001
Low physical activity group (%)	19.2	14.1	19.4	23.9	<0.001	19.7	19.6	18.0	<0.001
**Medical conditions**									
Obese (BMI > 30) (%)	18.6	15.9	17.4	23.6	<0.001	20.8	18.2	17.6	<0.001
Post-menopause in women (%)	41.3	34.6	41.7	54.5	<0.001	40.9	40.8	44.6	<0.001
Hypertension (%)	44.7	46.3	46.9	48.8	<0.001	46.4	46.9	48.0	0.002
Diabetes (%)	3.7	3.9	3.7	3.8	0.057	5.3	3.7	2.6	<0.001
High cholesterol (%)	82.3	81.6	83.0	81.5	<0.001	82.7	82.7	81.1	<0.001
**Dietary intake**									
Energy intake (MJ/day)^†^	8.69 ± 2.23	8.19 ± 2.06	8.35 ± 1.98	10.06 ± 2.49	<0.001	9.35 ± 2.39	8.26 ± 2.06	8.99 ± 2.29	<0.001
Energy density (kJ/g) ^†^	6.5 ± 1.6	4.8 ± 0.8	6.4 ± 0.8	8.4 ± 1.4	<0.001	7.1 ± 1.6	6.3 ± 1.5	6.4 ± 1.6	<0.001
Saturated fatty acids (%E)^†^	11.7 ± 3.2	9.7 ± 2.6	11.8 ± 2.8	13.4 ± 3.3	<0.001	14.4 ± 2.9	11.3 ± 2.7	10.0 ± 2.8	<0.001
Free sugars (%E)^†^	11.4 ± 5.2	8.8 ± 4.1	11.3 ± 4.5	14.5 ± 6.1	<0.001	7.6 ± 3.3	10.5 ± 3.6	17.3 ± 5.2	<0.001
Fiber (g/day)^†^	18.1 ± 6.2	23.3 ± 6.7	17.2 ± 5.0	15.2 ± 5.3	<0.001	18.2 ± 6.2	17.8 ± 6.0	18.5 ± 6.8	<0.001
Fiber density (g/MJ)^†^	2.1 ± 0.6	2.9 ± 0.6	2.1 ± 0.4	1.5 ± 0.4	<0.001	2.0 ± 0.6	2.2 ± 0.7	2.1 ± 0.7	<0.001

*Analysis of variance or chi-square test where appropriate. ^†^Plus-minus values are means ± standard deviation (SD)

### Associations between dietary pattern and outcomes

There were 4245 cases of incident CVD, 838 CVD deaths and 3629 deaths from all causes during 907,431 persons years of follow-up (7.8 years of median follow-up from baseline; 4.9 years from the last dietary assessment), respectively.

**Fig. 2:**
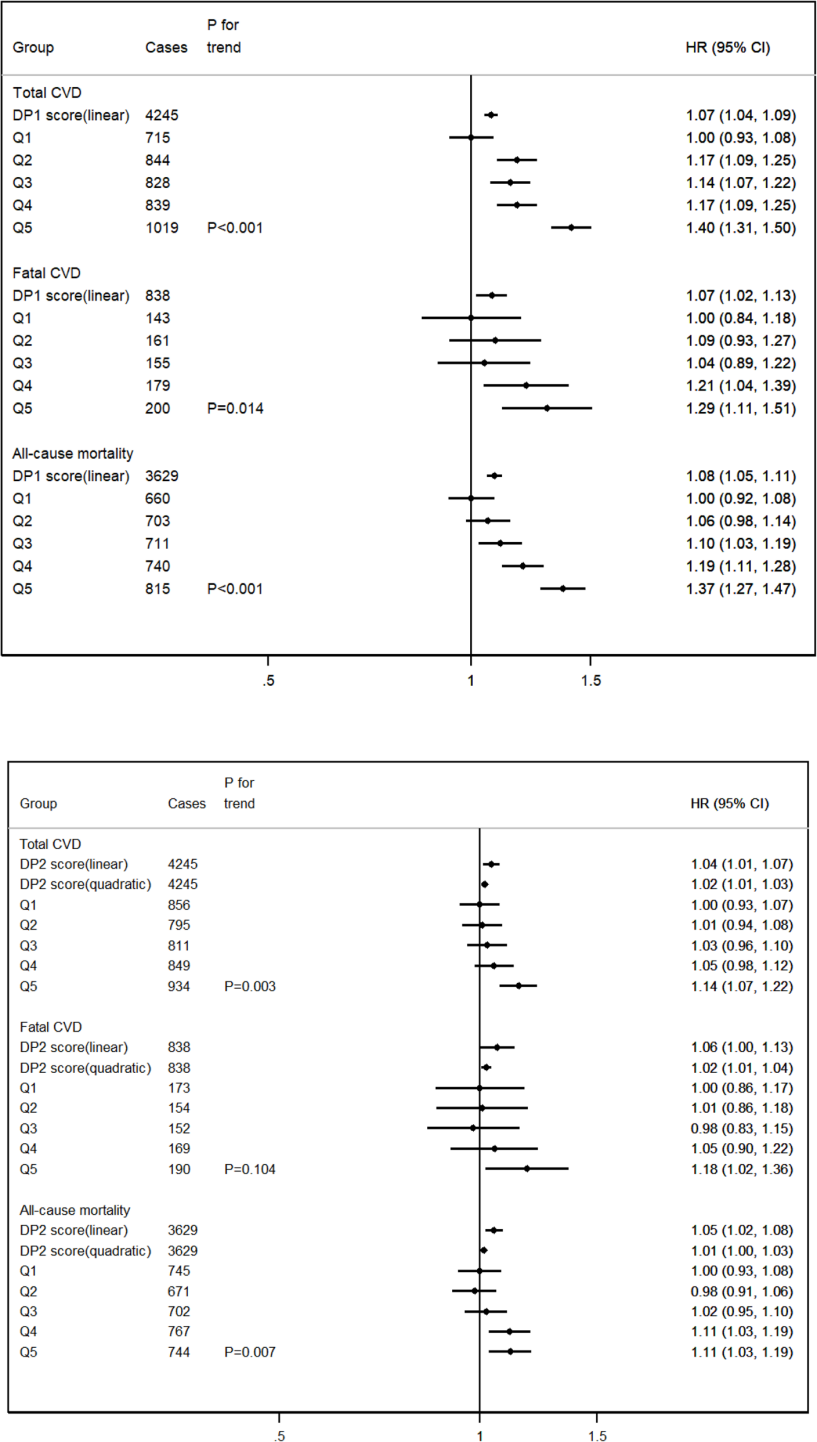
Prospective associations between dietary patterns and the risk of total CVD events and all-cause mortality (*n* = 116,806). Notes: All the models were stratified by sex and regions (England, Scotland, and Wales) and adjusted for ethnicity, socioeconomic status, behavioral risk factors, energy intake, and menopause in women. *Z*-scores for DP1 and DP2 were analyzed in mutually adjusted models to examine their independent associations with health outcomes. Adjusted HRs (hazard ratio) and confidence intervals (CI) of DP scores quintiles obtained using the floated absolute risk method of Cox proportional hazards regression, which enabled the comparisons across different quintiles of *z*-score. Trend tests were conducted by including the median score of each pattern quintile as a continuous variable in the models

We found a positive linear association for each standard deviation increase in dietary pattern 1 z-score and risk of total CVD (hazard ratio 1.07, 95% confidence interval 1.04 to 1.09), fatal CVD (1.07, 1.02 to 1.13), and all-cause mortality (1.08, 1.05 to 1.11) (Fig. 2, Additional file 1: Table S6). This association was also positive across dietary pattern 1 quintiles (*P*_for trend_ < 0.05). The nonlinear association between dietary pattern 2 z-scores and health outcomes was described by a quadratic model, total CVD (1.02, 1.01 to 1.03), fatal CVD (1.02, 1.01 to 1.04), and all-cause mortality (1.01, 1.00 to 1.03).

Analysis of splines also showed a linear association between dietary pattern 1 z-scores and total CVD, fatal CVD, and all-cause mortality. For dietary pattern 2, there was a non-linear association with total CVD, fatal CVD, and all-cause mortality (Additional file 1: Figure S6).

Sensitivity analysis excluding participants who had a CVD event within 2 years of completing their last 24-h online dietary assessment showed that the associations between dietary patterns and the risk of total and fatal CVD events and all-cause mortality were unchanged (Additional file 1: Table S7).

**Fig. 3:**
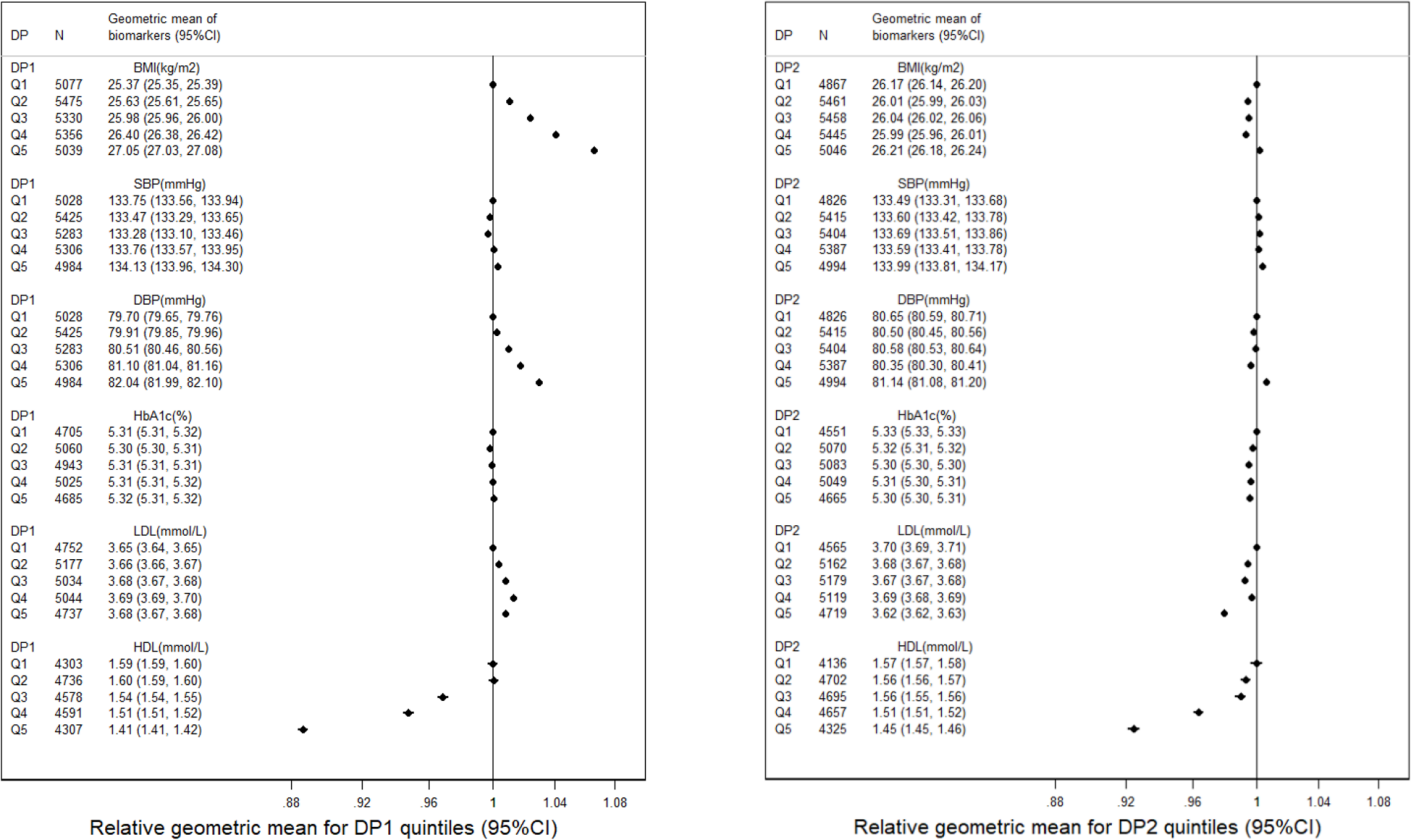
Baseline biomarkers by quintiles of dietary pattern scores among patients have both baseline and another dietary assessment during the follow-up (*N* = 26,277)

In cross-sectional analyses, people scoring high on dietary pattern 1 had increased BMI, DBP, and lower HDL at baseline, while people scoring high on dietary pattern 2 had generally no clinically meaningful differences on levels of their BMI or biomarkers except for a lower HDL (Fig. 3).

Mediation analysis found the association between the highest quintile of dietary pattern 1 and incident CVD was slightly attenuated following adjustment for the BMI group (Additional file 1: Figure S7), while the association between dietary pattern 2 and total CVD was not attenuated when adjusted for BMI group, hypertension, diabetes, and high cholesterol individually or simultaneously.

In subgroup analyses, a significantly higher risk of total CVD was observed among participants with dietary pattern 1 who were aged < 60 years (1.09, 1.05 to 1.13), or living with overweight (1.07, 1.03 to 1.11) or obesity (1.05, 1.00 to 1.10). A significantly greater association between dietary pattern 2 and total CVD risk was observed among women (1.02, 1.01 to 1.03), aged < 60 years (1.01, 1.00 to 1.03), and those with obesity (1.02, 1.00 to 1.04) (Additional file 1: Figure S8).

## Discussion

In this sample of middle-aged British adults, two principal dietary patterns explained 43% and 20% of the variance in specific nutrients, namely energy density, saturated fat, free sugars, and fiber, which are hypothesized to be on the pathway between the associations of food groups and CVD and all-cause mortality through their contribution to excess energy intake. In the primary pattern, greater consumption of chocolate and confectionery, butter, refined bread, and table sugar and preserves together with low intakes of fresh fruit, vegetables, and wholegrain foods was significantly associated with increased CVD and all-cause mortality. A second pattern was related to higher intakes of free sugars, predominately from sugar sweetened beverages, fruit juice, chocolate and confectionary, and table sugar and preserves but low in butter and higher fat cheese. The association of this dietary pattern with incident CVD and all-cause mortality was non-linear, with only evidence of increased risk for those with the highest dietary pattern *z*-scores. Exploratory analyses suggested the association observed with dietary pattern 1 was potentially mediated by excess weight.

RRR has not been widely used to identify dietary patterns and their associations with CVD risks. The first dietary pattern largely confirms previous studies reporting associations with a priori “Western” dietary patterns and the benefits of “Mediterranean” diets, and with a large body of data reporting the associations between individual food groups or nutrients and disease outcomes from prospective cohort studies in the USA and Europe [9, 11, 33, 34]. It is notable that people in the dietary pattern quintile with the lowest risk had mean intakes of energy from SFA of 9.7%, very close to the national and international recommendations, and free sugars accounted for 8.8% of total energy, below the World Health Organization (WHO) guidelines [35], though this level still exceeded the more stringent UK recommendations [36].

The second dietary pattern is more unusual and is characterized by higher intakes of sugar-sweetened beverages, fruit juice, and table sugar and preserves, together with lower intakes of high fat cheese and butter. This dietary pattern is striking because people in the highest quintile, with very high free sugars intake, otherwise followed other healthy behaviors, with higher physical activity, lower alcohol intake, and were less likely to smoke, and their intake of SFA met the recommended levels. People in the highest quintile for this dietary pattern had increased risks for CVD and all-cause mortality and consumed on average, 17.3% of dietary energy from free sugars, more than three times the UK dietary guideline, but only 10% SFA, which is the recommended level. While some previous research has shown that higher consumption of SSBs and other added sugars are associated with a higher risk of CVD [9, 37–40] and all-cause mortality [41], recent reviews of the evidence by the WHO [42], and by the UK Scientific Advisory Committee on Nutrition [36] did not identify a specific link between sugar intakes and total mortality.

### Implications of this research

This analysis supports dietary recommendations to limit particular foods groups, specifically chocolate and confectionery, butter and other animal fat spreads (primarily butter), low-fiber bread, sugar-sweetened beverages (SSBs), fruit juice, and table sugar and preserves. By identifying the food groups and dietary patterns which are associated with reduced risk of CVD or all-cause mortality, it adds useful details over and above nutrient recommendations to inform public health interventions to support people to achieve meaningful dietary changes to improve health. Promoting food-based dietary guidelines can help to reduce the conflict perceived by the public between recommendations to reduce saturated fat and free sugars, and considering foods within the context of an overall dietary patterns means that the recommendations are likely to be culturally appropriate.

The role of animal-based fat spreads such as butter is less clear since this is positively related to poor outcomes in dietary pattern 1 and negatively in dietary pattern 2. This reflects the ongoing debate about the role of saturated fatty acids from dairy products, rich in stearic acid, which appear to have less atherogenic potential than foods rich in other, longer chain, saturated fatty acids [11]. It implies that, at the present time, dietary advice to reduce saturated fatty acids should focus primarily on sources of saturated fat, such as chocolate and confectionery [43], and red and processed meat [44], which are consistently associated with adverse health outcomes. Overall, this study suggests that energy-dense diets with high intakes of free sugars but low intakes of vegetables, fruit, and fiber (dietary pattern 1), animal fats may contribute to an increased risk of CVD and mortality. However, a dietary pattern that is very high in free sugars, even if low in animal fats and SFA (dietary pattern 2), can also potentially increase CVD and mortality risk. However, there was no evidence to support the hypothesis that traditional CVD risk factors mediated the associations between dietary pattern 2 and CVD, and therefore, further research is needed to understand this association.

The strengths of this study include a large sample size and outcome ascertainment by linkage to medical records that minimized the loss to follow-up. Our estimates of dietary intake were consistent with official statistics from the UK NDNS (National Diet and Nutrition Survey, 2014/15-2015/16) [16], suggesting that our results could have moderate generalizability with the total UK adult population. We adjusted for multiple confounders and conducted sensitivity analyses to confirm the robustness of the derived dietary patterns and associations with CVD outcomes.

Unlike other forms of analysis of dietary patterns, RRR provides a link between the mechanistic evidence which is largely based on the effects of individual nutrients on disease processes and the natural mode of consumption of foods in the population [22]. This prospective study in a large cohort of people provides evidence to help inform food-based dietary guidelines which are sensitive to current patterns of consumption in the UK. By focusing on the food groups which have the highest or lowest factor loadings, public health planning can target the foods where changes in intake would be expected to yield the greatest improvement in health outcomes.

### Study limitations

A limitation of this study is that dietary intakes were measured by multiple 24-h online dietary assessments, which like all self-reported exposures, are subject to recall bias and misreporting, and are dependent on the accuracy of the food composition databases. In our analyses, dietary data from a minimum of two 24-h online dietary assessments was used to derive dietary patterns, to capture estimates which are closer to usual intake than a single measure [29, 45]. This approach was supported by our sensitivity analyses that derived identical dietary patterns when restricting the sample to individuals that provided 2, 3, 4, or 5 dietary questionnaires. Nevertheless, incident CVD in the few years following observations of diet reflects a lifetime’s exposure, and our measures of dietary pattern within a short follow-up period reflect only the most recent portion of this. Moreover, the CVD risk factors and biomarkers used in the cross-sectional and mediation analysis were only measured at baseline, which were 1 to 4 years before the dietary data collection. Finally, the dietary patterns identified may only be applicable to a UK population, since the combination of foods is likely to be culturally specific, but this method could be used to understand other cultural patterns of food consumption and their association with preventable disease. It is impossible to randomly allocate people to lifetime exposures to diet and so most nutritional epidemiology is observational, as was the case here. Uncertainty remains as to whether these dietary patterns are stable over time, although previous evidence using data from two time points suggests patterns were generally consistent over 18 years of follow-up [46]. Previous studies have reported that participants completing more dietary questionnaires tended to be older and more educated [47], which may limit the generalizability to the wider UK adult population.

## Conclusions

This analysis shows that diets high in chocolate confectionery, butter, refined bread, and table sugar and preserves, together with low intakes of fresh fruit, vegetables, and wholegrain foods, are associated with an increased risk of CVD and all-cause mortality, in part through a link to excess weight (dietary pattern 1). It also suggests that diets particularly high in sugar-sweetened beverages, fruit juice, table sugar and preserves (dietary pattern 2) may be an independent risk factor for premature mortality, and the mechanisms underpinning this association require further investigation. The present study helps identify specific foods and beverages which are major contributors to unhealthy dietary patterns and provides evidence to underpin food-based dietary advice to reduce health risks.

## Supplementary Information

**Additional file 1: Table S1.** Literature review on diet and CVD using reduced rank regression. **Table S2.** Food groups and their contents. **Table S3.** Derivation of variables used in analysis from the UK Biobank questionnaire and interviews. **Table S4.** Explained variation (%) in food intakes and response variables for each dietary pattern as assessed using reduced rank regression and correlation coefficient between dietary patterns and response variables. **Table S5.** Health outcomes and baseline characteristics of participants in two main dietary patterns. **Table S6.** Sequentially adjusted hazard ratios of CVD and all-cause mortality associated with dietary patterns. **Table S7.** Associations between dietary pattern scores and the risk of total and fatal CVD and all-cause mortality excluding people who had the event within two years after completing their last 24-h online dietary assessment. **Figure S1.** Participant flow chart of the study. **Figure S2.** Factor loadings for dietary patterns among people with 3+ times of 24-h online dietary assessments in the UK Biobank. **Figure S3.** Factor loadings for dietary patterns among people with 4+ times of 24-h online dietary assessments in the UK Biobank. **Figure S4.** Factor loadings for dietary patterns among people with 5 times of 24-h online dietary assessments in the UK Biobank. **Figure S5.** Factor loadings for dietary patterns excluding people who had the event within two years after completing their last 24-h online dietary assessment in the UK Biobank. **Figure S6.** HRs (95% CIs) of continuous dietary pattern scores for the risk of total and fatal CVD events and all-cause mortality. **Figure S7.** Relationships between dietary pattern scores in quintile 5 compared to quintile 1 and the risk of total CVD events, further adjusted for potential mediators of the associations. **Figure S8.** The association between dietary patterns and risk of total CVD by subgroups.

## Data Availability

The datasets generated/and or analyzed in the current study will be made available for bona fide researchers who apply to use the UK Biobank data set by registering and applying at http://www.ukbiobank.ac.uk/register-apply.

## Abbreviations

RRR: Reduced rank regression
CVD: Cardiovascular disease
HR: Hazard ratio
SBP: Systolic blood pressure
DBP: Diastolic blood pressure
HbA1c: Glycated hemoglobin A1c
LDL: Low-density lipoprotein
HDL: High-density lipoprotein
