# Supplementary material for: Associations between dietary patterns and the incidence of total and fatal cardiovascular disease and all-cause mortality in 116,806 individuals from the UK Biobank: a prospective cohort study
Source: BMC Med. 2021 Apr 22;19:83. doi: 10.1186/s12916-021-01958-x (PMC8061025; doi:10.1186/s12916-021-01958-x)
Supplement: Supplementary file 1 — Additional file 1: Table S1. Literature review on diet and CVD using reduced rank regression. Table S2. Food groups and their contents. Table S3. Derivation of variables used in analysis from the UK Biobank questionnaire and interviews. Table S4. Explained variation (%) in food intakes and response variables for each dietary pattern as assessed using reduced rank regression and correlation coefficient between dietary patterns and response variables. Table S5. Health outcomes and baseline characteristics of participants in two main dietary patterns. Table S6. Sequentially adjusted hazard ratios of CVD and all-cause mortality associated with dietary patterns. Table S7. Associations between dietary pattern scores and the risk of total and fatal CVD and all-cause mortality excluding people who had the event within two years after completing their last 24-h online dietary assessment. Figure S1. Participant flow chart of the study. Figure S2. Factor loadings for dietary patterns among people with 3+ times of 24-h online dietary assessments in the UK Biobank. Figure S3. Factor loadings for dietary patterns among people with 4+ times of 24-h online dietary assessments in the UK Biobank. Figure S4. Factor loadings for dietary patterns among people with 5 times of 24-h online dietary assessments in the UK Biobank. Figure S5. Factor loadings for dietary patterns excluding people who had the event within two years after completing their last 24-h online dietary assessment in the UK Biobank. Figure S6. HRs (95% CIs) of continuous dietary pattern scores for the risk of total and fatal CVD events and all-cause mortality. Figure S7. Relationships between dietary pattern scores in quintile 5 compared to quintile 1 and the risk of total CVD events, further adjusted for potential mediators of the associations. Figure S8. The association between dietary patterns and risk of total CVD by subgroups. [file 12916_2021_1958_MOESM1_ESM.docx]

**Index**

**Additional Table S1**: Literature review on diet and CVD using reduced rank regression

**Additional Table S2:** Food groups and their contents

**Additional Table S3:** Derivation of variables used in analysis from the UK Biobank questionnaire and interviews

**Additional Table S4:** Explained variation (%) in food intakes and response variables for each dietary pattern as assessed using reduced rank regression and correlation coefficient between dietary patterns and response variables (N=116 806)

**Additional Table S5:** Health outcomes and baseline characteristics of participants in two main dietary patterns (N=116 806)

**Additional Table S6**: Sequentially adjusted hazard ratios of CVD and all-cause mortality associated with dietary patterns (N=116 806)

**Additional Table S7:** Associations between dietary pattern scores and the risk of total and fatal CVD and all-cause mortality excluding people who had the event within two years after completing their last 24-h online dietary assessment (N=115 532)

**Additional Fig S1:** Participant flow chart of the study

**Additional Fig S2:** Factor loadings for dietary patterns characterized by energy density (kJ/g), saturated fat (%total energy), free sugar (%total energy) and fiber density (g/MJ) calculated by using reduced rank regression among people with 3+ times of 24-h online dietary assessments in the UK Biobank (N=72 912)

**Additional Fig S3:** Factor loadings for dietary patterns characterized by energy density (kJ/g), saturated fat (%total energy), free sugar (%total energy) and fiber density (g/MJ) calculated by using reduced rank regression among people with 4+ times of 24-h online dietary assessments in the UK Biobank (N=33 760)

**Additional Fig S4:** Factor loadings for dietary patterns characterized by energy density (kJ/g), saturated fat (%total energy), free sugar (%total energy) and fiber density (g/MJ) calculated by using reduced rank regression among people with 5 times of 24-h online dietary assessments in the UK Biobank (N=5 403)

**Additional Fig S5:** Factor loadings for dietary patterns characterized by energy density (kJ/g), saturated fat (%total energy), free sugar (%total energy) and fiber density (g/MJ) calculated by using reduced rank regression excluding people who had the event within two years after completing their last 24-h online dietary assessment in the UK Biobank (N=115 532)

**Additional Fig S6:** HRs (95% CIs) of continuous dietary pattern scores for the risk of total and fatal CVD events and all-cause mortality (n=116 806)

**Additional Fig S7:** Relationships between dietary pattern scores in quintile 5 compared to quintile 1 and the risk of total CVD events, further adjusted for potential mediators of the associations (n=116 806)

**Additional Fig S8:** The association between dietary patterns and risk of total CVD by subgroups (N=116 806)

**Additional Table S1: Literature review on diet and CVD using reduced rank regression**

Search terms used on PubMed, Embase (up to 14th February 2020):

("dietary pattern" or "diet") AND ("reduced rank regression" or "RRR") AND ("mortality" or "death" or "survival" or "cardiovascular disease" or "CVD" or "stroke" or "coronary heart disease" or “CHD” or "congestive heart failure" or “CHF” or “ischemic heart disease” or “IHD” or "coronary artery disease" or "CAD")

| Author | Study name (date of recruitment) | Study participants (location, N, age) | Method to identify dietary patterns | Diet measurement/collecting | Response variables | Main RRR-derived dietary pattern and (% variance explained) | Outcomes | Methods for estimating associations with outcomes | Association between RRR dietary patterns (DP) and outcomes |
| --- | --- | --- | --- | --- | --- | --- | --- | --- | --- |
| LONGITUDINAL COHORT STUDIES | | | | | | | | | |
| Lamichhane, et al. (2014) | The SEARCH for Diabetes in Youth Study (2001) | USA, n=1153, aged 10-19 years with T1DM | RRR | Modified version of the Block Kid’s FFQ (85-item)/Baseline diet data | Triglycerides, low density lipoprotein-cholesterol, systolic blood pressure, haemoglobin A1c, C-reactive protein and waist circumference | DP1: High intakes of sugar-sweetened beverages (SSB) and diet soda, eggs, potatoes and high-fat meats and low intakes of sweets/desserts and low-fat dairy (4.3%) | Arterial stiffness (AS): augmentation index (AIx-75), pulse wave velocity (PWV), and brachial distensibility (BrachD) | Linear regression analyses | DP1 and AIx-75: +(*), DP1 and PWV: +, BrachD : - |
| He´roux, et al. (2009) | Aerobics Center Longitudinal Study (ACLS) (1987-1999) | USA, n=13621, aged 20-84 years | RRR | 3-day food records/Baseline diet data | BMI, blood pressure, total cholesterol, high-density lipoprotein (HDL)- cholesterol, triglycerides, uric acid, white blood cell count and fasting glucose | DP1: High consumption of processed and red meat, white potato products, non-whole grains, added fat and reduced consumption of non-citrus fruits (76.5%) | CVD mortality, overall mortality | Cox regression analyses | DP1 and CVD mortality: +, DP1 and All-cause mortality: +(*) |
| Biesbroek, et al. (2015) | European Prospective Investigation into Cancer–Netherlands (1993-1997) | Netherlands, n=34644, aged 20–70 years | RRR, PCA, RF-CTA, KCA | FFQ(178-item)/diet data collected during the year preceding enrolment | BMI, SBP, and ratio of total to HDL cholesterol | Western, prudent and traditional patterns (N/A) | Incident coronary artery disease (CAD) and stroke | Cox regression analyses | Traditional DP and CAD: +(*), Western DP and CAD: +(*), Prudent DP and CAD: -(*), Traditional DP and stroke: +, Western DP and stroke: +, Prudent DP and stroke: -(*) |
| Johns, et al. (2015) | Swedish Obese Subjects study (SOS) (1987-2001) | Sweden, n=2037, obese individuals aged 37-60 years | RRR | SOS dietary questionnaire/measured ten times/all diet data was used | Dietary energy density, saturated fat intake, and fiber density | DP1: Energy-dense, high-saturated-fat, and low- fiber DP (54%) | Cardiometabolic risk factors and incident CVD | Cox regression analyses | DP1 and Cardiometabolic risk factors: +(*), DP1 and CVD: + |
| Meyer, et al. (2011) | MONItoring of Trends and Determinants in Cardiovascular Diseases’ (MONICA) (1984-1995) | German, N=981, men aged 45–64 years | RRR, PLS and PCR | 7-day dietary record/Baseline diet data | C-reactive protein, interleukin (IL)-6 and IL-18 | DP1: High intakes of meat, soft drinks and beer and low intakes of vegetables, fresh fruit, chocolates, cake, pastries, wholemeal bread, cereals, muesli, curd, condensed milk, cream, butter, nuts, sweet bread spread and tea (5.01%) | Incident CHD and all-cause mortality | Cox regression analyses | DP1 and incident CHD: +, DP1 and CHD mortality: +, DP1 and all-cause mortality: +(*) |
| McNaughton, et al. (2009) | The Whitehall II study (1991-1993) | UK, n=7314, aged 35-55 years | RRR | FFQ (127-item)/Baseline diet data | Total cholesterol, HDL cholesterol and triglycerides | DP1: Diet characterized by high consumption of white bread, fried potatoes, sugar in tea and coffee, burgers & sausages, soft drinks, and low consumption of French dressing and vegetables (4.2%) | Incident CHD | Cox regression analyses | DP 1 and incident CHD: +(*) |
| Drake, et al. (2018) | The Malmö Diet and Cancer Study (MDCS) (1991-1996) | Sweden, n=4071, aged 45–67 years | RRR | 7-d food record, 168-item quantitative diet history questionnaire, diet history interview /Baseline diet data | Waist circumference, TAG, HDL- and LDL-cholesterol, systolic and diastolic blood pressure, fasting glucose and insulin | DP1: 'Western' (2.5%), DP2: 'Drinker' (0.7%) | Incident metabolic syndrome (iMetS) | Multivariable linear regression analyses | DP1 and iMetS: +(*), DP2 and iMetS: + |
| Nazari, et al. (2018) | The Multi-Ethnic Study of Atherosclerosis (MESA) (2000) | USA, n=5468, aged 45–84 years | RRR, PCR, and PLS | FFQ(120-item) /Baseline diet data | BMI, fibrinogen, interleukin-6, and low-density lipoprotein cholesterol. | DP1: high in fats and oils, poultry, non-diet soda, processed meat, tomatoes, legumes, chicken, tuna and egg salad, and fried potatoes and low in dark-yellow and cruciferous vegetables (13.97%) | Incidence stroke | Cox regression analyses | DP 1 and stroke incidence: +(*) |
| Liese, et al. (2010) | The prospective Insulin Resistance Atherosclerosis Study (IRAS) (1992-1994) | USA, n=802, aged 40-69 years | RRR | FFQ(114-item) /Baseline diet data | Plasminogen activator inhibitor 1(PAI-1) and fibrinogen | DP1: higher intakes of less healthful foods and lower intakes of more healthful foods (8.7%) | Common (CCA) and internal carotid artery (ICA) intimal medial thickness (IMT) | Multi-variable adjusted linear regression | DP 1 and CCA IMT: +(*), DP 1 and ICA IMT: + |
| Drogan, et al. (2007) | The EPIC-Potsdam Study (1994-1998) | German, n=26238, aged 35-65 years | RRR | Self-administered FFQ (148-item) /Baseline diet data | Weight change | DP: high consumption of whole-grain bread, fruits, fruit juices, grain flakes and/or cereals, and raw vegetables, and a low consumption of processed meat, butter, high-fat cheese, margarine, and meat other than poultry (N/A) | CVD morbidity and mortality | Cox regression analyses | DP and CVD morbidity: +, DP and CVD mortality: -(*) |
| Hoffmann, et al. (2005) | The European Prospective Investigation into Cancer and Nutrition (EPIC)-Potsdam Study (1994-1998) | German,n=9356, aged ≥60 years | PCA amd RRR | FFQ(148-item)/Baseline diet data | The percentages of energy from saturated fat, monounsaturated fat, polyunsaturated fat, protein and carbohydrates | DP1: high consumption of all types of meat, butter, sauces and eggs (30.8%). DP 2: high consumption of legumes, poultry, fish and margarine (15.9%) | All-cause mortality | Cox regression analyses | DP 1 and all-cause mortality: +(*), DP 2 and all-cause mortality: - |
| Mazidi, et al. (2019) | The US National Health and Nutrition Examination Survey (NHANES) database (1999-2010) | USA, n=20256, aged ≥ 18 years | RRR | 24 h recalls | 37micro and macro nutrients | Dietary Hyperuricaemia Score (DUS) (14 micro/macronutrients) and Dietary Atherogenic Score (DAS) (23 micro/macronutrients): high consumption of uricaemia and atherogenic diets (N/A) | All-cause and cause-specific mortality | Cox regression analyses | DUS and All-Cause Mortality: +(*), DUS and CVD Mortality: +(*), DUS and Cancer Mortality: +(*), DAS and All-Cause Mortality: +(*), DAS and CVD Mortality: +(*), DAS and Cancer Mortality: +(*) |
| CASE-CONTROL STUDIES | | | | | | | | | |
| Hoffmann, et al. (2004) | The Coronary Risk Factors for Atherosclerosis in Women (CORA) Study (1997-2001) | German, n=455, women aged 30-80 years | RRR | FFQ(146-item) /Baseline diet data | LDL and HDL cholesterol, C-reactive protein, Lipoprotein(a), C-peptide | DP1: high intakes of meat, margarine, poultry, and sauce and low intakes of vegetarian dishes, wine, vegetables, and whole-grain cereals (7.8%) | Incident CAD | Conditional logistic regression | DP 1 and incident CAD: +(*) |
| Weikert, et al. (2005) | The Coronary Risk Factors for Atherosclerosis in Women (CORA) Study (1997-2001), the European Prospective Investigation into Cancer and Nutrition (EPIC)-Potsdam Study (1994-1998) | German, n=27250, aged 30-80 years | RRR | FFQ(148-item)/Baseline diet data | Plasma concentrations of Hcy, folate, and vitamin B-12 | DP1: Whole-grain bread, fresh fruit, olive oil, mushrooms, cruciferous vegetables, wine, and nuts contributed the most positively and fried potatoes the most negatively to a dietary pattern (8.9%) | Incident CHD | Conditional logistic regression analyses and Cox regression analyses | DP1 and CHD: -(*) |
| Seah, et al. (2019) | Singapore Chinese Health Study(1993-1998) | Singapore, n=45411, aged 45-74 years | RRR | Interviewer administered semi-quantitative FFQ (165-item) /Baseline diet data | 19 fatty acid biomarkers | DP1: High in soy, vegetables, fruits, tea, tomato products, bread, fish, margarine and dairy, and low in rice, red meat, coffee, alcohol, sugar-sweetened beverages, and eggs (N/A) | Incident T2D and CAD mortality | Cox regression analyses | DP1 and type 2 Diabetes: -(*), DP1 and CAD: -(*) |
| Ehsani, et al. (2016) | The Tehran Lipid and Glucose study (TLGS) (1999-2001) | Iran, n=220, women aged 18-45 years | RRR | FFQ (168-item) /The fourth follow-up examination | Visceral Adiposity Index (VAI) | VAI dietary pattern (25%) | Cardiometabolic profiles | Logistic and linear regression analyses | Control: DP and Abdominal obesity: -, DP and Visceral adiposity dysfunction: +(*), DP and Hypertension: +, DP and High FPG: +(*), DP and IGT+IFG: +(*), DP and Hypercholesterolemia: +(*), DP and Hypertriglyceridemia: +(*), DP and High LDL cholesterol: +, DP and Low HDL cholesterol: +(*). PCOS: DP and Abdominal obesity: -, DP and Visceral adiposity dysfunction: +(*), DP and Hypertension: +, DP and High FPG: +, DP and IGT+IFG: +, DP and Hypercholesterolemia: -, DP and Hypertriglyceridemia: +, DP and High LDL cholesterol: -, DP and Low HDL cholesterol: +. |
| CROSS-SECTIONAL STUDIES | | | | | | | | | |
| Nettleton, et al. (2007) | The Multi-Ethnic Study of Atherosclerosis (MESA)(2000) | USA, n=5089, aged 45–84 years | RRR and PCA | Modified-Block style FFQ/Baseline diet data | CRP, IL-6, fibrinogen, and homocysteine | DP1: High in total and saturated fat and low in fiber and micronutrients (5%) | Coronary artery calcium (CAC) and intima media thickness(IMT) | Multivariable logistic regression analyses and linear regression analyses | DP1 and IMT: +(*), DP1 and CAC: +(*) |
| Livingstone, et al. (2017) | Australian Health Survey (2011–2013) | Australia, n=4908, aged≥19 years | RRR | 24-h dietary recalls/two times/mean values | Dietary energy density, fiber density and total sugar intake for obesity, Na:K, SFA:PUFA and fiber density for hypertension | Obesity-DP1: high fiber density and sugars and low DED (36.4%). Obesity-DP2: high sugars and low fiber density (18.0%). Hypertension-DP1: high Na:K and SFA:PUFA (33.3%). Hypertension-DP2: low Na:K and SFA:PUFA (12.6%) | Prevalent overweight/obesity, hypertension | Logistic, Poisson and linear regression analyses | Obesity-DP1 and obesity: -(*), Obesity-DP2 and obesity: +(*), Hypertension-DP1 and hypertension:+(*), Hypertension-DP2 and hypertension: - |
| Centritto, et al. (2009) | Moli-sani project (2005-2008) | Italy, n=7646, aged≥35 years | RRR and PFA | The Italian validated EPIC FFQ(188-item) /Baseline diet data | Total, HDL and LDL cholesterol, triglycerides, systolic and diastolic blood pressure, blood glucose and CRP | "Pasta and Meat" pattern (6%) | CVD risk profile and C-reactive protein (CRP) | Multivariable linear regression analysis | "Pasta and Meat" pattern and CVD risk profile: +(*), "Pasta and Meat" pattern and CRP: +(*) |
| DiBello, et al. (2008) | Costa Rican study (1994–2004) | Costa Rica, n=3574 | RRR, PLS and PCA | Modified version of the Willett questionnaire (135-item)/Baseline diet data | Adipose tissue levels of α-linolenic and trans-fatty acids and dietary intakes of saturated fat, fiber, and folate | Vegetable pattern: high intakes of lean chicken, vegetables, fruit, and polyunsaturated oil (N/A) | First incident myocardial infarction | Conditional logistic regression analyses | Vegetable pattern and myocardial infarction: -(*) |
| +: positive association. -: negative association. (*): statistically significant. N/A: didn’t report related information. Abbreviations: DP: dietary pattern, RRR: reduced rank regression, FFQ: food frequency questionnaire, AS: Arterial stiffness, AIx-75: augmentation index, PWV: pulse wave velocity, BrachD: brachial distensibility, BMI: body mass index, HDL: high-density lipoprotein, LDL: low-density lipoprotein, CVD: cardiovascular disease, PCA: principal component analysis, KCA: k-means cluster analysis, RF-CTA: random forest with classification tree analysis, PFA: principal factor analysis, PLS: partial least-squares regression, CAD: coronary artery disease, CHD: incidence of coronary heart disease, IL-6: interleukin-6, IL-18: interleukin-18, iMetS: incident metabolic syndrome, CCA: common carotid artery, ICA: internal carotid artery, IMT: intima media thickness, DUS: dietary hyperuricaemia score, DAS: dietary atherogenic score, VAI: visceral adiposity index, PCOS: polycystic ovary syndrome, FPG: fasting plasma glucose  IGT: impaired glucose tolerance, IFG: impaired fasting glucose, CAC: coronary artery calcium, CRP: C-reactive protein, SFA: saturated fat intake, PUFA: polyunsaturated fatty acids | | | | | | | | | |

502 536 participants recruited between 2006 to 2010

**116 806** participants, including:

**4 245** total CVD cases

**838** fatal CVD cases

**3 629** all-cause mortality cases

**Exclusion criteria:**

- People without any validated 24-h online dietary assessment (n=291 514)
- People without 2 or more validated 24-h online dietary assessment data (n=84 166)
- People with CVD events that occurred before baseline assessment (n=6 422)
- People with CVD events that occurred before completing the last assessment (n=1 337)
- Pregnancy (n=108)

Excluded samples with missing values

(Total=1 084):

- Response variables (n=10)
- Population demographics
  - Ethnicity(n=40)
- Socioeconomic status
  - Townsend(n=138)
  - Education(n=326)
- Behavioral risk factors
  - Smoking status (n=198)
- Medical conditions
  - BMI (n=239)
  - Diabetes (n=132)
  - High Cholesterol (n=1)

Excluded samples with implausible energy intake (Total=1 099):

- Under-reporters (n=980)
- Over-reporters (n=119)

**Additional Figure S1: Participant flow chart of the study**

**Additional Table S2: Food groups and their contents**

| Food group | Food items |
| --- | --- |
| High-fat milk and cream | Whole milk >3.6 g fat per 100 g |
|  | Cream |
|  | Full fat yogurt |
| Low-fat milk | Semi skimmed milk >1 g fat per 100 g |
|  | Skimmed milk |
|  | Low fat yogurt |
| Non-dairy milk | Rice/oat milk |
|  | Soya |
| High fat cheese | High fat cheese |
| Low fat cheese | Low fat cheese |
| Olive oil | Olive oil (drizzling/dunking) |
| Poultry | Poultry |
| Red meat | Pork |
|  | Beef |
|  | Lamb |
|  | Other meat |
| Processed meat | Processed meat |
| Oily fish | Oily fish |
| Other fish | Shellfish |
|  | White fish & tinned tuna |
| Coated or breaded meat and fish | Breaded/battered chicken |
|  | Breaded/battered fish |
| Egg & egg dishes | Egg & egg dishes |
| Meat substitutes | Meat substitutes - vegetarian |
|  | Meat substitutes - soy |
| Legumes & pulses | Legumes & pulses |
| Vegetables | Raw salad |
|  | Green leafy/cabbages |
|  | Root vegetables |
|  | Tomatoes |
|  | Allium vegetables |
|  | Other vegetables, including mushrooms, fruiting and mixed veg |
|  | Peas/sweetcorn |
| Boiled or baked potatoes | Potatoes/Sweet potatoes (baked/boiled) |
|  | Mashed potatoes |
| Fried or roast potatoes | Fried/roast potatoes |
| Low-fiber bread | White bread |
| High-fiber bread | Wholemeal bread |
|  | Mixed (50/50), brown & seeded |
| Other bread products | Other bread |
|  | Savoury crackers |
|  | Grain dishes - added fat |
|  | Samosa, pakora |
| Fresh fruit | Citrus |
|  | Berries |
|  | Apples & pears |
|  | Other fruit |
| Dried and stewed fruit | Dried fruit |
|  | Stewed fruit |
| Pasta and rice | White pasta & rice |
|  | Sushi |
| Wholemeal pasta, brown rice & other wholegrains | Wholemeal pasta, brown rice & other wholegrains |
| Pizza | Pizza |
| Nuts & seeds | Salted nuts & seeds |
|  | Unsalted nuts & seeds |
| Fruit juice | Fruit juice |
| Coffee and tea | Coffee, caffeinated |
|  | Coffee, decaffeinated |
|  | Tea |
|  | Tea, decaffeinated |
| Sugar-sweetened beverages & other sugary drinks | Sugar-sweetened beverages & other sugary drinks |
| Low/non sugar SSBs | Low/non sugar SSBs |
| Alcoholic drinks(Wine, beer, spirits) | White wine |
|  | Red wine |
|  | Fortified wine |
|  | Beer & Cider |
|  | Spirits |
| Water/Sparkling water | Water/Sparkling water |
| Milk-based and powdered drinks | Milk-based and powdered drinks |
| Soups | Soups |
| Sauces & condiments (high fat) | Sauces & condiments (high fat) |
| Sauces & condiments (low fat) | Sauces & condiments (low fat) |
| Milk-based desserts | Milk-dairy desserts |
|  | Soy desserts and yogurt |
| Grain-based desserts | Other desserts & cakes & pastries |
| Table sugars & preserves | Table sugars & preserves |
| Chocolate and confectionery | Chocolate confectionary |
|  | Other sweets |
| High-fiber breakfast cereals | Bran cereal |
|  | Biscuit cereal |
|  | Porridge |
| Other breakfast cereals | Oat cereal (sugar) |
|  | Muesli |
|  | Other cereal (sugar) |
| Crisps and savoury snacks | Savoury snacks |
| Vegetable side dishes and dips | Vegetable side dishes |
|  | Vegetable dips |
| Low animal fat spread | Reduced fat animal fat spread |
| Butter and other animal fat spreads | Butter and other animal fat spreads |
| Lower plant-based fat spread | Reduced fat plant-based fat spread |
| Normal plant-based fat spread | Plant-based fat spread |
| Nut-based spread | Nut-based spread |

**Additional Table S3: Derivation of variables used in analysis from the UK Biobank questionnaire and interviews**

| Variables | Categories used in analysis | UK Biobank variable used (question ID) and source |
| --- | --- | --- |
| *Health outcomes* | | |
| Total CVD cases | No  Yes | Hospital admission data: ICD-10: coronary heart disease (CHD; I20-I25), congestive heart failure or cardiomyopathy (CHF; I50, I50.1, 150.9, I11.0, I13.0, I13.2, I42, I43.1), and total stroke (I60-I64) |
| Fatal CVD cases | No  Yes | Death registry data linked to the UK Biobank: ICD-10, I00-I25, I27-I88, and I95-I99 (ID: 40001 / 40002 ) |
| All-cause mortality cases | No  Yes | Death registry data linked to the UK Biobank (ID: 40001 / 40002 ) |
| *Sociodemographic characteristics* | | |
| Sex | Men  Women | Sex (ID: 31)* |
| Age | Age | Age at recruitment (ID: 21022)* |
| Ethnicity | Others  White | Ethnic background (ID: 21000)* |
| Townsend index | Quintiles 1-5 (high index indicates most deprivation) | Townsend index (ID: 189)* |
| Education | Higher degree (college or university degree, or professional qualifications)  Any school degree (A levels, AS levels, O levels, GCSEs or CSEs)  Vocational qualifications (NVQ, HND or HNC)  Other (none of the above qualifications) | Qualifications (ID: 6138)^†^ |
| *Behavioral risk factors* | | |
| Smoking status | Never  Current  Previous | Smoking status (ID: 20116)^†^ |
| Energy intake | kcal | Participants with implausible energy intakes were excluded. We used the individualized method to estimate the ratio of reported energy intake to estimated energy requirement (EER) (EI:EER). Individual EERs were calculated with the use of Schofield Equation. A 95% CI was calculated to classify individuals as plausible reporters (EI:EER within the 95% CI), over-reporters (EI:EER >95%CI) or under-reporters (EI:EER <95% CI). |
| Physical activity (IPAQ MET scores) | Low (<600 metabolic equivalent (MET)-minutes per week)  Moderate (≥600 and <3000 metabolic equivalent (MET)-minutes per week)  High (≥ 3000 metabolic equivalent (MET)-minutes per week) | Number of days/week of vigorous physical activity 10+ minutes (ID: 904)^†^; Duration of vigorous activity (ID: 914)^†^  Number of days/week of moderate physical activity 10+ minutes (ID: 884)^†^; Duration of moderate activity (ID: 894)^†^  Number of days/week walked 10+ minutes (ID: 864)^†^; Duration of walks (ID: 874)^†^ |
| *Medical conditions* | | |
| BMI | Underweight (<18.5)  Healthy weight(18.5 to <25)  Overweight (25 to <30)  Obese(30+) | BMI (ID: 21001 )^‡^ |
| Menopause | N/A (if respondents are men )  No  Yes (if responded to questions saying they have experienced the menopause) | Had menopause (women only) (ID: 2724)^†^ |
| Hypertension | No  Yes (if diagnosed by doctor, had an SBP >140mmHg, DBP >90mmHg or taking medication for blood pressure) | Vascular/heart problems diagnosed by doctor (high blood pressure is one response) (ID: 6150)^†^  Systolic blood pressure, automated reading / manual reading (ID: 4080 / 93)^‡^  Diastolic blood pressure, automated reading / manual reading (ID: 4079 / 94)^‡^  Medication for cholesterol, blood pressure or diabetes (men) (ID: 6177)^†^; Medication for cholesterol, blood pressure, diabetes, or take exogenous hormones (women) (ID: 6153)^†^ |
| Diabetes | No  Yes (if diagnosed by doctor or taking medication for diabetes or HbA1c≥6.5%) | Diabetes diagnosed by doctor (ID: 2443)^†^  Medication for cholesterol, blood pressure or diabetes (men) (ID: 6177)^†^; Medication for cholesterol, blood pressure, diabetes, or take exogenous hormones (women) (ID: 6153)^†^; Glycated haemoglobin (HbA1c) (ID: 30750) ^#^ |
| High Cholesterol | No  Yes (if taking cholesterol lowering medication or LDL cholesterol>3 mmol/L or total cholesterol>5 mmol/L ) | Medication for cholesterol, blood pressure or diabetes (men) (ID: 6177)^†^; Medication for cholesterol, blood pressure, diabetes, or take exogenous hormones (women) (ID: 6153)^†^; blood lipids (ID: 30690,30760)^#^ |
| Note: *Recruitment questions, ^†^Touchscreen questions, ^‡^Physical measurements, ^§^24-h online dietary assessment questionnaire, ^#^biological samples | | |

| Additional Table S4: Explained variation (%) in food intakes and response variables for each dietary pattern as assessed using reduced rank regression and correlation coefficient between dietary patterns and response variables (N= 116 806) | | | | | | | | | | |
| --- | --- | --- | --- | --- | --- | --- | --- | --- | --- | --- |
| Dietary patterns | Explained variation (%) | | | | | | Correlation coefficient | | | |
|  | Food intakes (total) | Response variables (total) | Energy density (kJ/g) | Saturated fat acids (%E) | Free sugars(%E) | Fiber density (g/MJ) | Energy density (kJ/g) | Saturated fat(%E) | Free sugars(%E) | Fiber density (g/MJ) |
| 1 | 3.8 | 43 | 65 | 23 | 22 | 61 | 0.81*** | 0.42*** | 0.40*** | -0.77*** |
| 2 | 2.1 | 20 | 1 | 22 | 54 | 0 | -0.15** | -0.49*** | 0.68*** | 0.06*** |
| 3 | 2.5 | 10 | 5 | 23 | 7 | 5 | -0.33*** | 0.37*** | 0.15*** | 0.37*** |
| 4 | 2.3 | 4 | 9 | 0 | 0 | 9 | 0.30*** | 0.03*** | 0.06*** | 0.26*** |
| Note: %E, proportion of total energy intake. ***:P<0.001, **:P<0.01. | | | | | | | | | | |

| **Additional Table S5: Health outcomes, baseline characteristics and dietary intake of participants in two main dietary patterns (N=116 806)** | | | | | | | | | | |  |
| --- | --- | --- | --- | --- | --- | --- | --- | --- | --- | --- | --- |
|  | | Total  (N=116806) | **Dietary pattern 1** | | | | **Dietary pattern 2** | | | | |
|  | |  | Quintile 1  (N=23362) | Quintile 3  (N=23361) | Quintile 5  (N=23361) | *P*  *value** | Quintile 1  (N=23362) | Quintile 3  (N=23361) | Quintile 5  (N=23361) | *P value** | |
| ***Total CVD cases***^†^ | | 4245 | 715 | 828 | 1019 | <0.001 | 856 | 811 | 934 | 0.007 | |
| ***Fatal CVD cases***^†^ | | 838 | 143 | 155 | 200 | 0.018 | 173 | 152 | 190 | 0.217 | |
| ***All-cause mortality cases*** | | 3629 | 660 | 711 | 815 | 0.001 | 745 | 702 | 744 | 0.075 | |
| ***Demographics*** | |  |  |  |  |  |  |  |  |  | |
| Sex (%) | |  |  |  |  | <0.001 |  |  |  | <0.001 | |
| Women | | 57.3 | 71.3 | 59.9 | 36.6 |  | 56.3 | 61.6 | 48.9 |  | |
| Men | | 42.7 | 28.7 | 40.1 | 63.4 |  | 43.7 | 38.4 | 51.1 |  | |
| Age(year) ^‡^ | | 55.9±7.8 | 57.2±7.3 | 56.1±7.7 | 53.9±8.1 | <0.001 | 55.9±7.7 | 56.1±7.8 | 55.1±8.1 | <0.001 | |
| Ethnicity (%) | |  |  |  |  | 0.212 |  |  |  | <0.001 | |
| Whites | | 96.7 | 96.5 | 96.8 | 96.6 |  | 97.6 | 97.2 | 94.6 |  | |
| ***Socioeconomic status*** | |  |  |  |  |  |  |  |  |  | |
| Townsend index (quintiles) | |  |  |  |  | <0.001 |  |  |  | <0.001 | |
| Q1 | | 20.1 | 20.2 | 20.7 | 18.5 |  | 19.5 | 20.6 | 19.3 |  | |
| Q3 | | 20.0 | 19.9 | 20.6 | 19.9 |  | 20.0 | 20.3 | 19.9 |  | |
| Q5 | | 19.9 | 19.3 | 18.6 | 22.5 |  | 20.8 | 18.8 | 21.4 |  | |
| Education group (%) | |  |  |  |  |  |  |  |  |  | |
| Vocational qualification (NVQ, HND or HNC) | | 12.5 | 10.5 | 12.4 | 16.1 | <0.001 | 12.0 | 11.9 | 13.8 | <0.001 | |
| Any school degree (A-level, AS-level, O-level, GCSE, CSE) | | 29.4 | 27.1 | 29.2 | 32.1 |  | 29.1 | 29.9 | 29.2 |  | |
| Higher degree (college, university or professional degree/qualification) | | 52.1 | 56.9 | 52.7 | 44.9 |  | 53.5 | 52.0 | 50.6 |  | |
| ***Behavioral risk factors*** | |  |  |  |  |  |  |  |  |  | |
| Smoking status (%) | |  |  |  |  | <0.001 |  |  |  | <0.001 | |
| Never | | 58.2 | 60.7 | 59.8 | 53.0 |  | 54.7 | 58.7 | 60.4 |  | |
| Previous | | 35.0 | 35.5 | 34.7 | 34.3 |  | 36.9 | 35.3 | 32.5 |  | |
| Current | | 6.8 | 3.8 | 5.5 | 12.6 |  | 8.5 | 6.0 | 7.1 |  | |
| Physical Activity group (%) | |  |  |  |  | <0.001 |  |  |  | <0.001 | |
| Low | | 19.2 | 14.1 | 19.4 | 23.9 |  | 19.7 | 19.6 | 18.0 |  | |
| Moderate | | 45.1 | 43.0 | 46.1 | 44.2 |  | 45.8 | 45.4 | 43.3 |  | |
| High | | 35.8 | 42.9 | 34.6 | 31.9 |  | 34.5 | 35.0 | 38.8 |  | |
| ***Medical conditions*** | |  |  |  |  |  |  |  |  |  | |
| BMI group (%)^†^ | |  |  |  |  | <0.001 |  |  |  | <0.001 | |
| Underweight (<18.5) | | 0.6 | 0.9 | 0.5 | 0.5 |  | 0.7 | 0.5 | 0.5 |  | |
| Healthy weight(18.5 to <25) | | 40.2 | 45.5 | 41.5 | 32.2 |  | 39.1 | 40.8 | 39.8 |  | |
| Overweight (25 to <30) | | 40.6 | 37.7 | 40.7 | 43.6 |  | 39.4 | 40.5 | 42.1 |  | |
| Obese (30+) | | 18.6 | 15.9 | 17.4 | 23.6 |  | 20.8 | 18.2 | 17.6 |  | |
| Post-menopause in women (%) | | 41.3 | 34.6 | 41.7 | 54.5 | <0.001 | 40.9 | 40.8 | 44.6 | <0.001 | |
| Hypertension (%) | | 44.7 | 46.3 | 46.9 | 48.8 | <0.001 | 46.4 | 46.9 | 48.0 | 0.002 | |
| Diabetes (%) | | 3.7 | 3.9 | 3.7 | 3.8 | 0.057 | 5.3 | 3.7 | 2.6 | <0.001 | |
| High cholesterol (%) | | 82.3 | 81.6 | 83.0 | 81.5 | <0.001 | 82.7 | 82.7 | 81.1 | <0.001 | |
| ***Dietary intake*** | |  |  |  |  |  |  |  |  |  | |
| Energy intake(MJ/day) ^‡^ | | 8.69±2.23 | 8.19±2.06 | 8.35±1.98 | 10.06±2.49 | <0.001 | 9.35±2.39 | 8.26±2.06 | 8.99±2.29 | <0.001 | |
| Energy density (kJ/g) ^‡^ | | 6.5±1.6 | 4.8±0.8 | 6.4±0.8 | 8.4±1.4 | <0.001 | 7.1±1.6 | 6.3±1.5 | 6.4±1.6 | <0.001 | |
| Saturated fatty acids (%E) ^‡^ | | 11.7±3.2 | 9.7±2.6 | 11.8±2.8 | 13.4±3.3 | <0.001 | 14.4±2.9 | 11.3±2.7 | 10.0±2.8 | <0.001 | |
| Free sugars (%E) ^‡^ | | 11.4±5.2 | 8.8±4.1 | 11.3±4.5 | 14.5±6.1 | <0.001 | 7.6±3.3 | 10.5±3.6 | 17.3±5.2 | <0.001 | |
| Fiber (g/day) ^‡^ | | 18.1±6.2 | 23.3±6.7 | 17.2±5.0 | 15.2±5.3 | <0.001 | 18.2±6.2 | 17.8±6.0 | 18.5±6.8 | <0.001 | |
| Fiber density (g/MJ) ^‡^ | | 2.1±0.6 | 2.9±0.6 | 2.1±0.4 | 1.5±0.4 | <0.001 | 2.0±0.6 | 2.2±0.7 | 2.1±0.7 | <0.001 | |
| ***Main food groups*** | |  |  |  |  |  |  |  |  |  | |
| Chocolate and confectionery | | 11.8±21.3 | 5.9±12.4 | 9.7±15.9 | 23.1±33.4 | <0.001 | 8.2±15.7 | 10.4±17.8 | 19.0±30.8 | <0.001 | |
| Butter and other animal fat spreads | | 5.1±8.9 | 1.9±4.8 | 4.3±7.2 | 10.5± 12.4 | <0.001 | 12.2±12.6 | 3.5±6.4 | 2.1±5.1 | <0.001 | |
| Low-fiber bread | | 28.0±42.1 | 9.2±21.9 | 22.4± 32.5 | 60.6±58.3 | <0.001 | 36.7±50.2 | 25.3±39.0 | 25.6±40.8 | <0.001 | |
| Sugar-sweetened beverages | | 86.6±165.4 | 44.4±106.0 | 73.9±134.4 | 162.0±245.2 | <0.001 | 30.3± 76.9 | 57.4±104.4 | 218.5±266.8 | <0.001 | |
| Fruit juice | | 109.2±137.7 | 102.7±140.2 | 113.1±135.7 | 108.8±145.2 | <0.001 | 51.6± 86.7 | 91.3± 107.4 | 206.1±190.0 | <0.001 | |
| Table sugar and preserves | | 8.6±12.8 | 5.2±8.2 | 7.4±10.0 | 15.2±19.1 | <0.001 | 5.8±9.2 | 7.1±10.2 | 14.6±18.2 | <0.001 | |
| High fat cheese and butter | | 14.9±18.2 | 11.0±15.4 | 14.6±16.9 | 19.5±22.5 | <0.001 | 28.1± 24.6 | 12.1±14.1 | 8.0±12.5 | <0.001 | |
| Fresh fruit | | 197.3±153.2 | 342.6±181.5 | 178.8±112.0 | 98.1±97.6 | <0.001 | 171.5± 143.6 | 200.9± 148.2 | 216.2± 169.3 | <0.001 | |
| Vegetables | | 191.2±146.2 | 325.7±185.5 | 171.0±106.3 | 106.8±92.1 | <0.001 | 196.2±148.3 | 191.6±140.7 | 184.9±156.2 | <0.001 | |
| High-fiber breakfast cereals | | 43.6±66.3 | 75.9±84.8 | 40.1±60.1 | 20.3± 42.8 | <0.001 | 35.7±61.7 | 45.7±66.3 | 46.8±70.1 | <0.001 | |
| **Note:**  *. Analysis of variance or chi2 test where appropriate. ^†^. Abbreviations: CVD: cardiovascular disease. NVQ: national vocational qualification. HND: higher national diploma. HNC: higher national certificate. GCSE: general certificate of secondary education. CSE: comprehensive sex education. BMI: body mass index. ^‡^. Plus-minus values are means ± standard deviation (SD). | | | | | | | | | | |  |

| 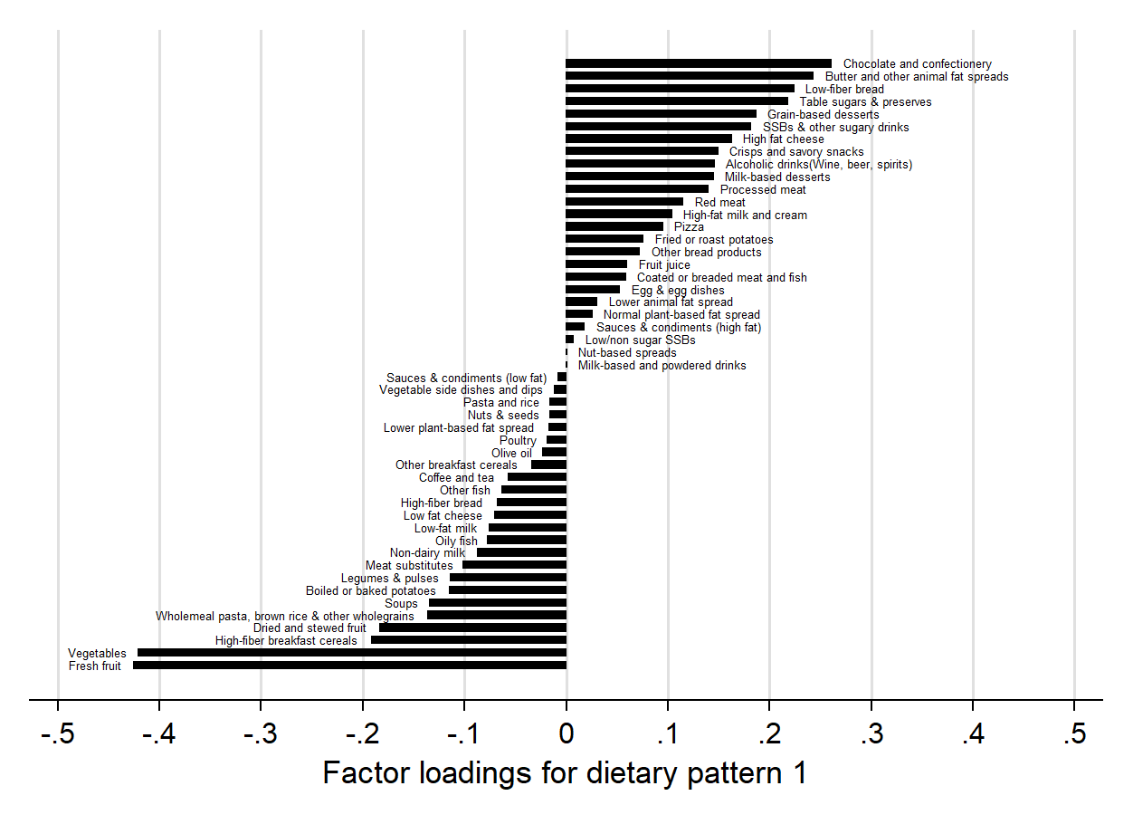 |
| --- |
| 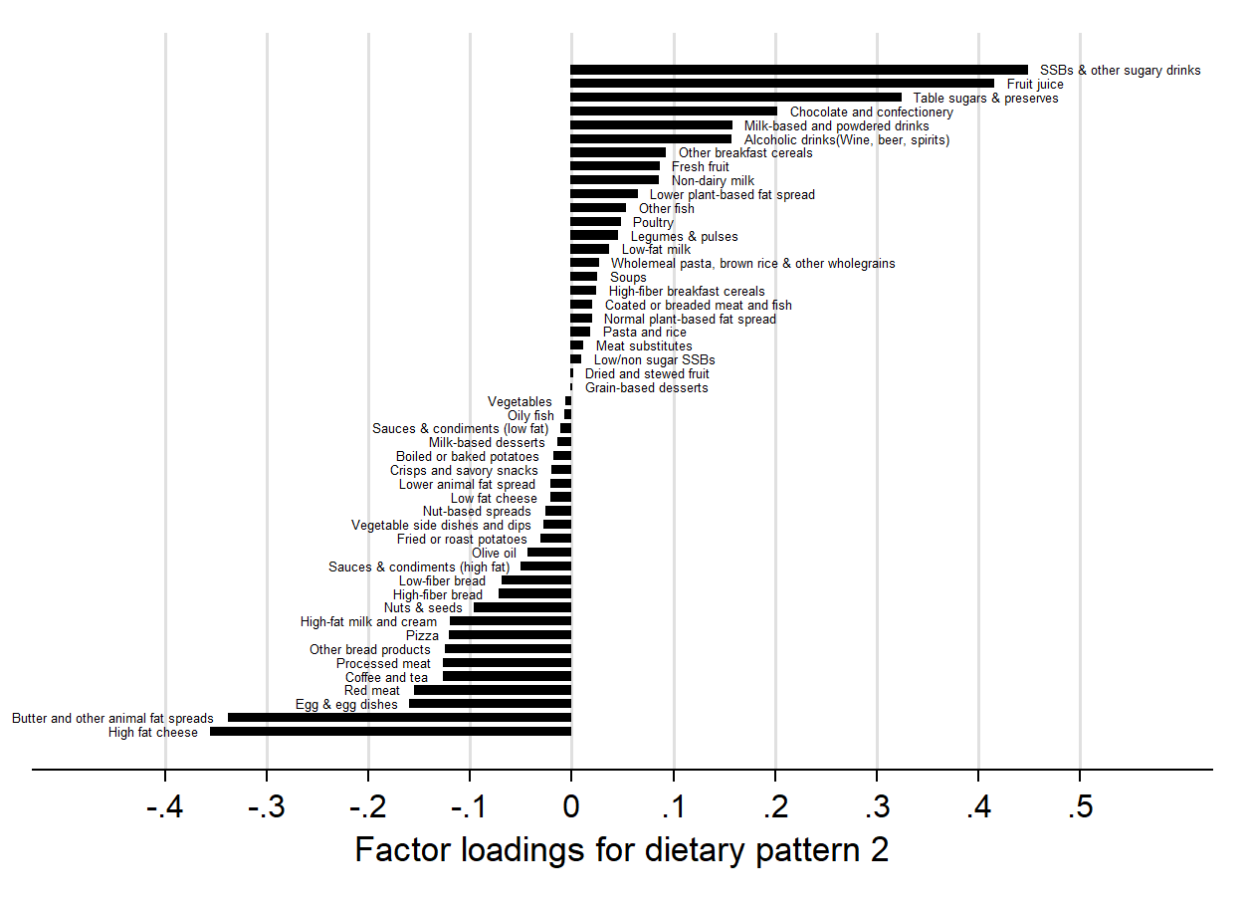 |
| **Additional Figure S2: Factor loadings for dietary patterns characterized by energy density (kJ/g), saturated fat acids (%total energy), free sugar (%total energy) and fiber density (g/MJ) calculated by using reduced rank regression among people with 3 or more 24-h online dietary assessments in the UK Biobank (N=72 912)** |


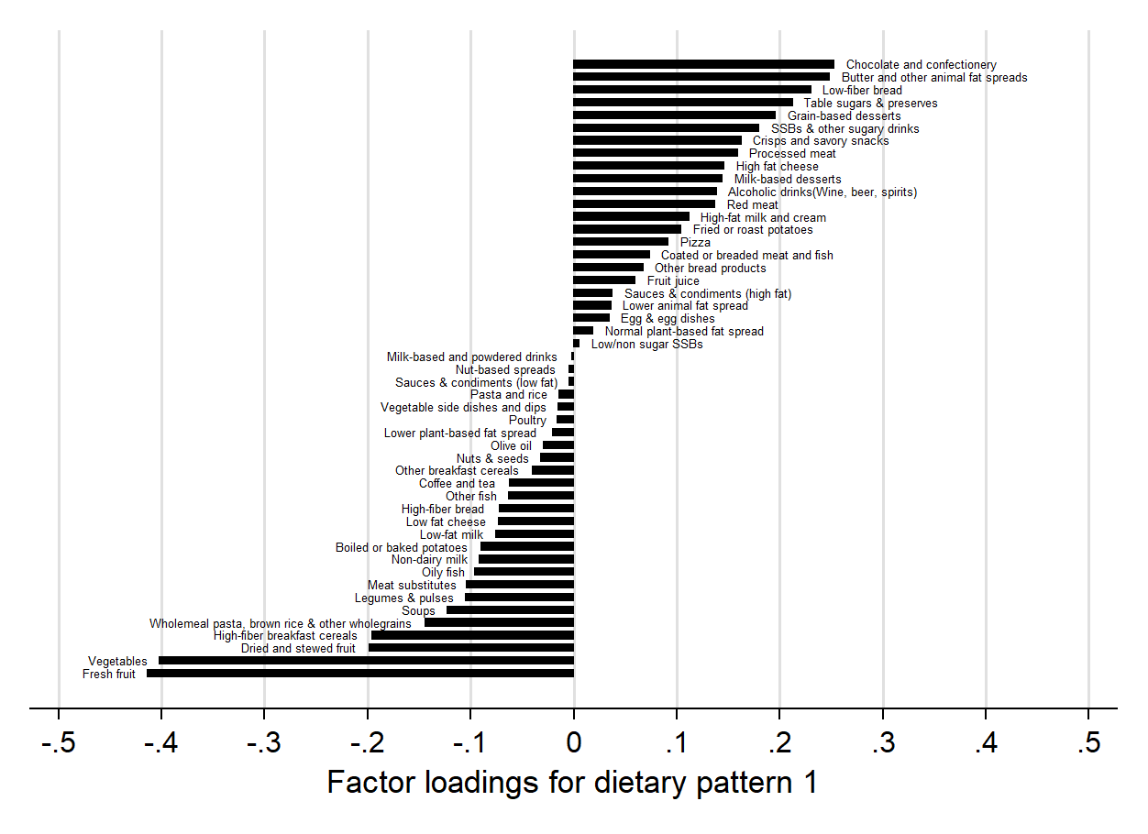


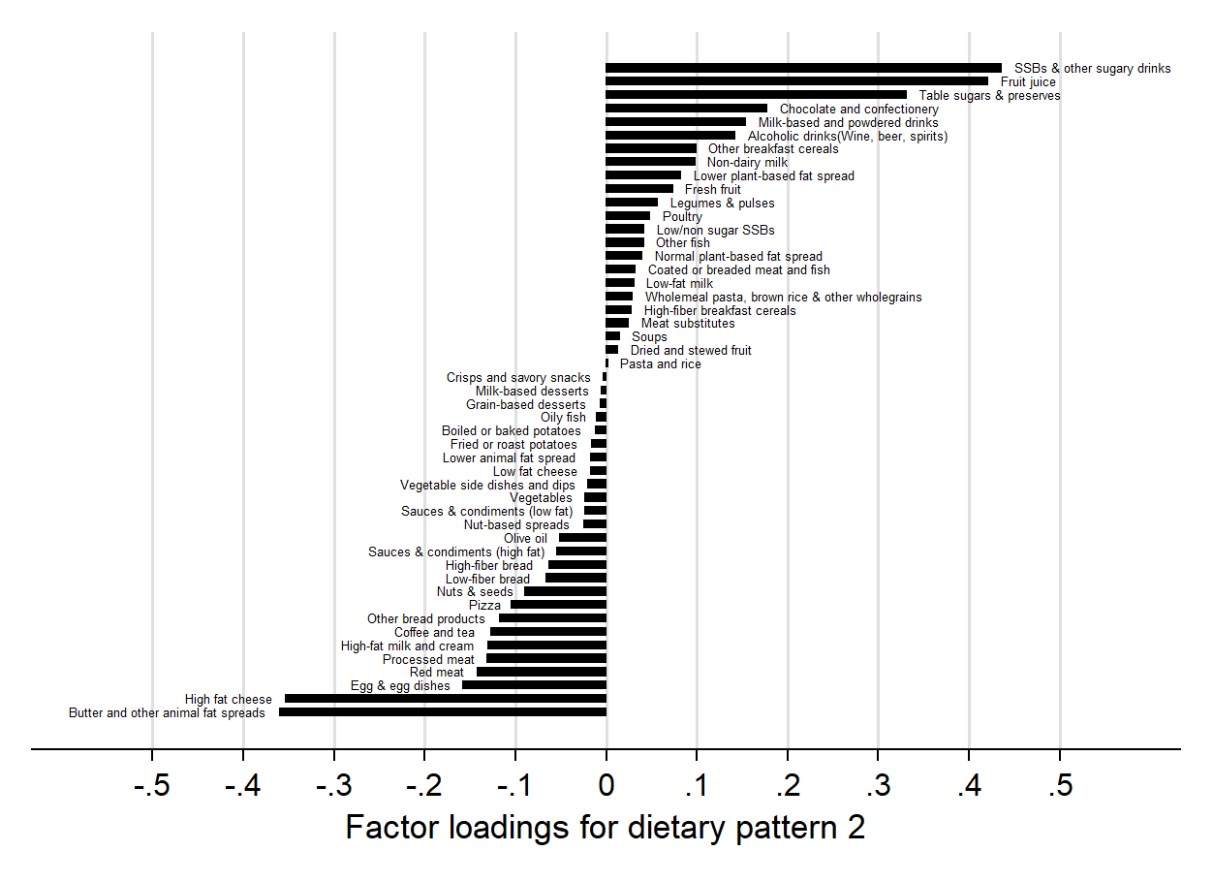


**Additional Figure S3: Factor loadings for dietary patterns characterized by energy density (kJ/g), saturated fat acids (%total energy), free sugar (%total energy) and fiber density (g/MJ) calculated by using reduced rank regression among people with 4 or more 24-h online dietary assessments in the UK Biobank (N=33 760)**


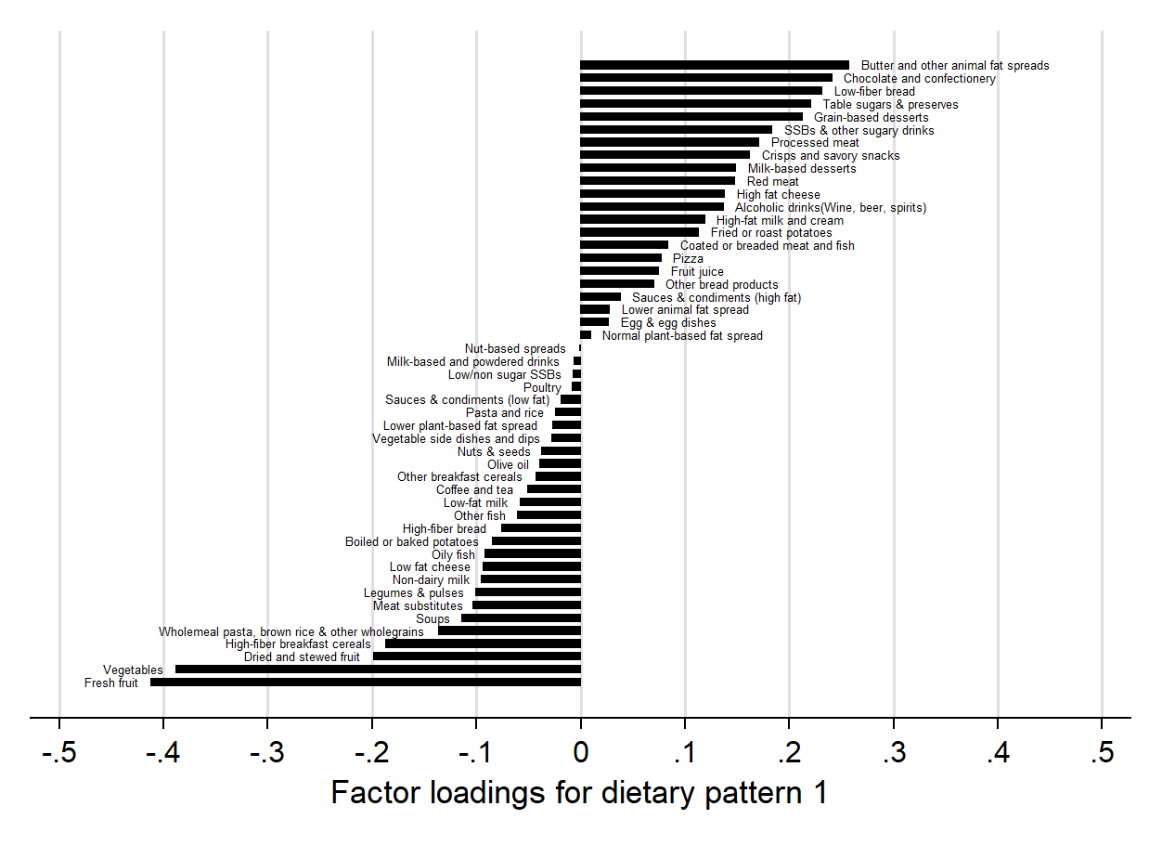


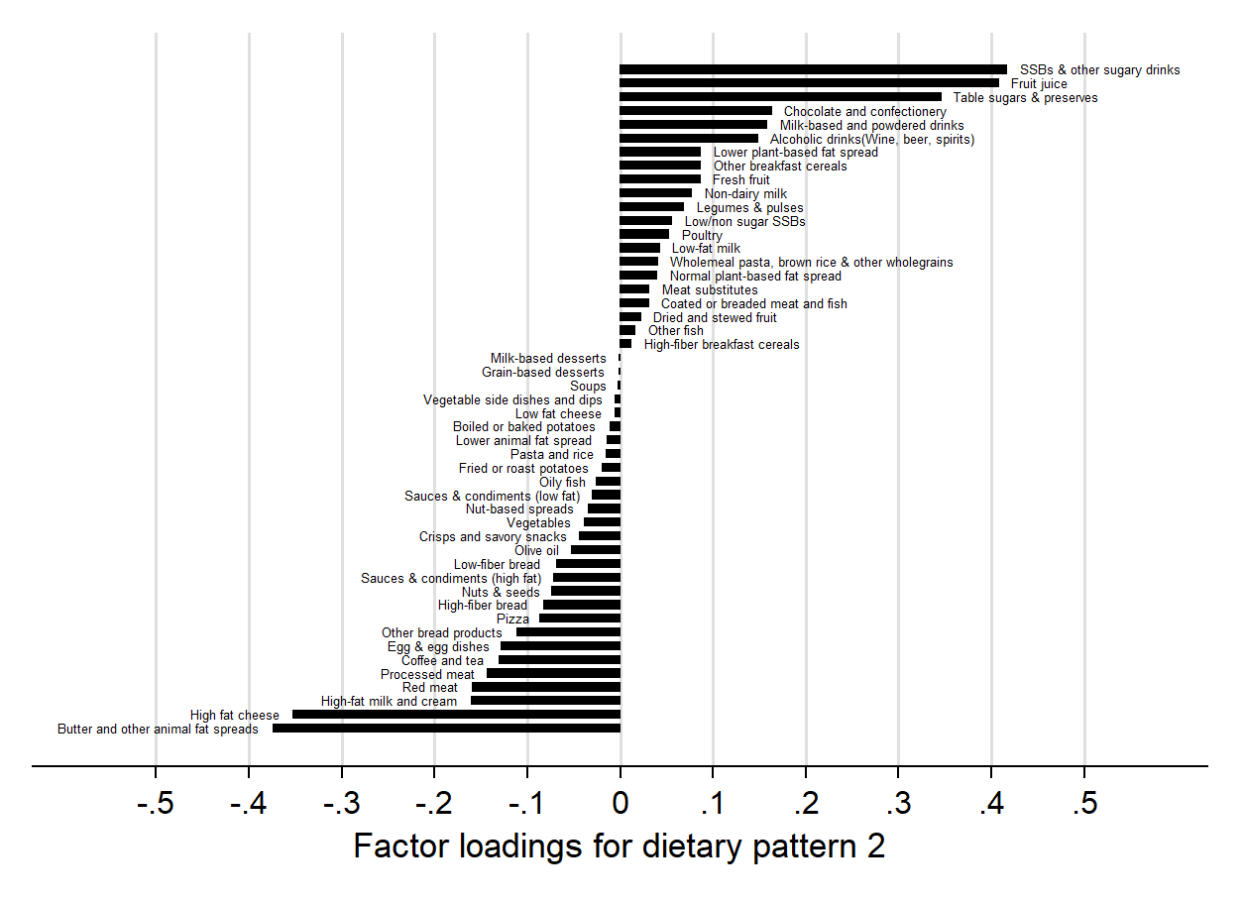


**Additional Figure S4: Factor loadings for dietary patterns characterized by energy density (kJ/g), saturated fat acids (%total energy), free sugar (%total energy) and fiber density (g/MJ) calculated by using reduced rank regression among people with five 24-h online dietary assessments in the UK Biobank (N=5 403)**


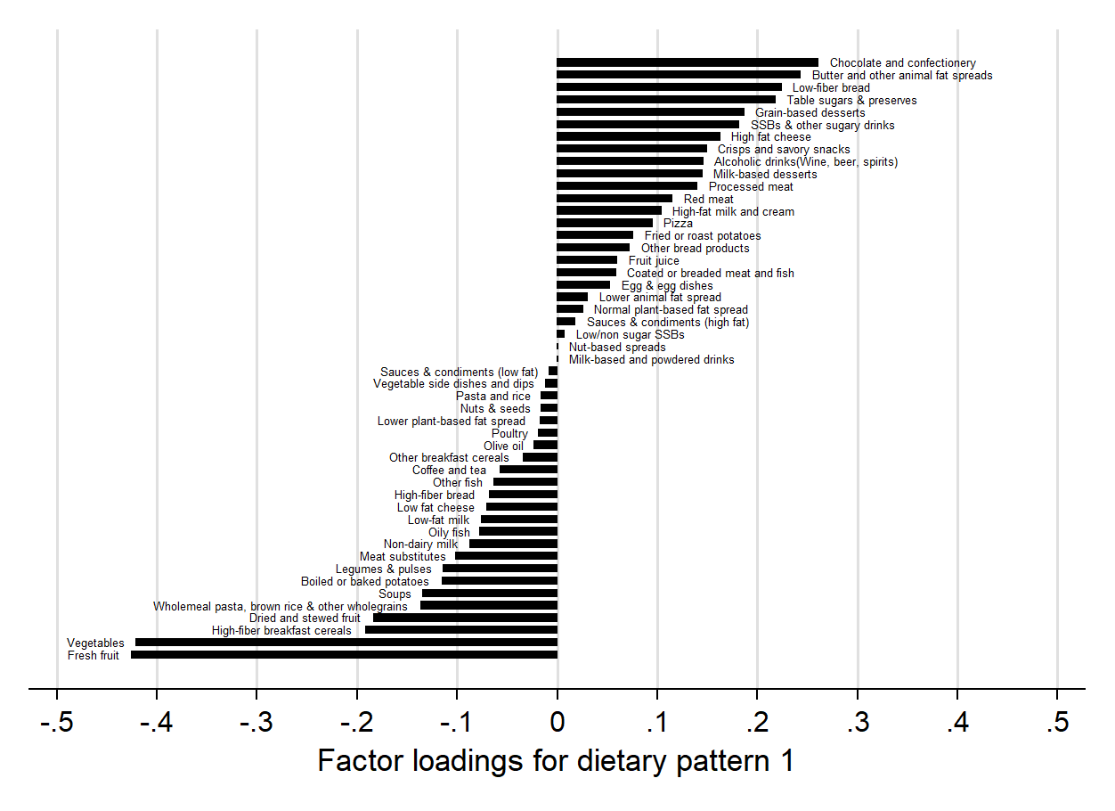


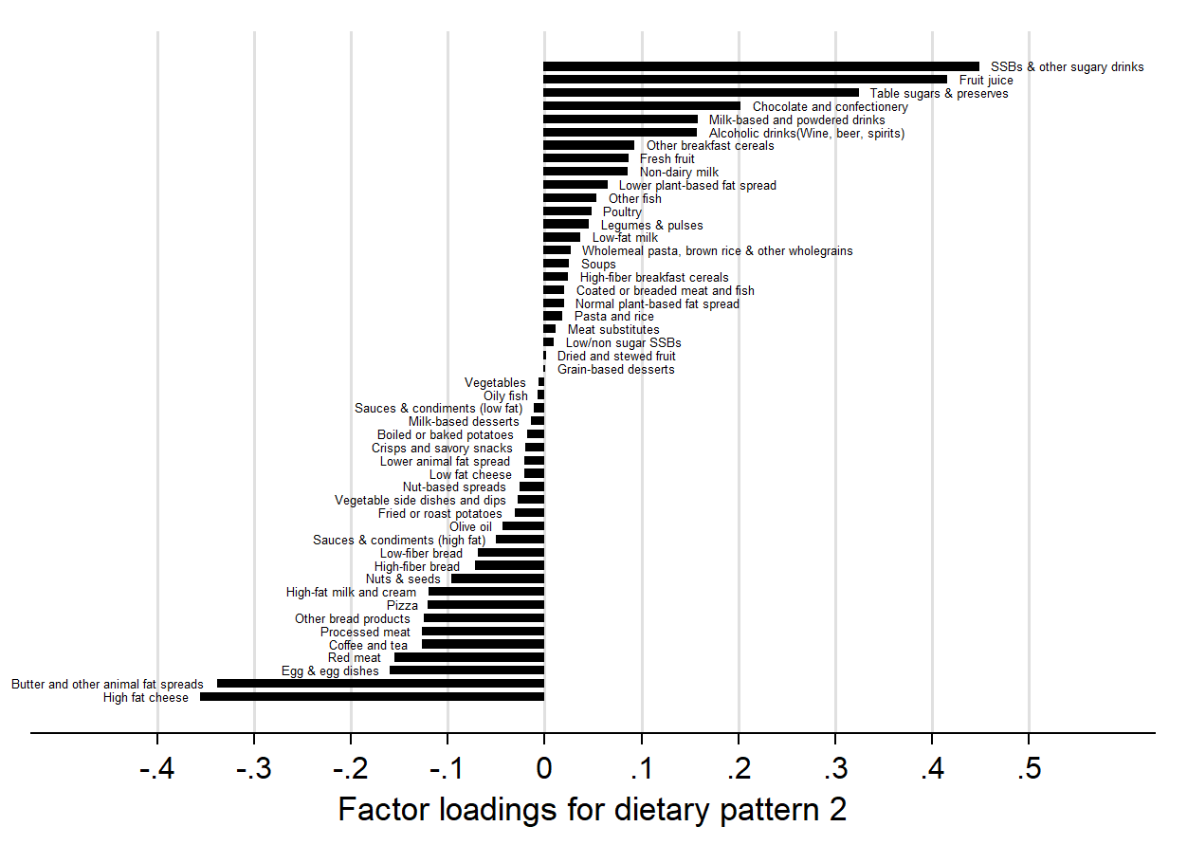


**Additional Figure S5: Factor loadings for dietary patterns characterized by energy density (kJ/g), saturated fat acids (%total energy), free sugar (%total energy) and fiber density (g/MJ) calculated by using reduced rank regression excluding people who had the event within two years after completing their last 24-h online dietary assessment in the UK Biobank (N=115 532)**

| Additional Table S6: Sequentially adjusted hazard ratios of CVD and all-cause mortality associated with dietary patterns (N=116 806) | | | | | | |
| --- | --- | --- | --- | --- | --- | --- |
|  | Group | No. events | Age adjusted, sex, and regions  stratified | + Socio-demographics* | + Behavioral risk factors ^†^ | +Energy intake, menopause in women ^‡^ |
| Total CVD cases | | | | | | |
| Dietary pattern 1 | Linear | 4245 | 1.09(1.06,1.11) | 1.08(1.06,1.11) | 1.07(1.04,1.09) | 1.07(1.04,1.09) |
|  | Q1 | 715 | 1.00(0.93,1.08) | 1.00(0.93,1.08) | 1.00(0.93,1.08) | 1.00(0.93,1.08) |
|  | Q2 | 844 | 1.18(1.10,1.27) | 1.18(1.10,1.26) | 1.17(1.09,1.25) | 1.17(1.09,1.25) |
|  | Q3 | 828 | 1.17(1.09,1.25) | 1.16(1.09,1.25) | 1.14(1.07,1.22) | 1.14(1.07,1.22) |
|  | Q4 | 839 | 1.21(1.13,1.29) | 1.20(1.13,1.29) | 1.17(1.09,1.25) | 1.17(1.09,1.25) |
|  | Q5 | 1019 | 1.50(1.40,1.60) | 1.48(1.39,1.58) | 1.40(1.31,1.49) | 1.40(1.31,1.50) |
|  | Non-linearity test  (p-value) ^§^ | | <0.001 | 0.009 | 0.003 | 0.002 |
| Dietary pattern 2 | Linear | 4245 | 1.04(1.02,1.07) | 1.04(1.01,1.07) | 1.04(1.01,1.07) | 1.04(1.01,1.07) |
|  | Quadratic | 4245 | 1.02(1.01,1.03) | 1.02(1.01,1.03) | 1.02(1.01,1.03) | 1.02(1.01,1.03) |
|  | Q1 | 856 | 1.00(0.93,1.07) | 1.00(0.93,1.07) | 1.00(0.93,1.07) | 1.00(0.93,1.07) |
|  | Q2 | 795 | 1.01(0.94,1.08) | 1.01(0.94,1.08) | 1.01(0.94,1.08) | 1.01(0.94,1.08) |
|  | Q3 | 811 | 1.03(0.96,1.10) | 1.03(0.96,1.10) | 1.03(0.96,1.10) | 1.03(0.96,1.10) |
|  | Q4 | 849 | 1.05(0.98,1.12) | 1.05(0.98,1.12) | 1.05(0.99,1.13) | 1.05(0.98,1.12) |
|  | Q5 | 934 | 1.15(1.08,1.23) | 1.14(1.07,1.21) | 1.15(1.08,1.22) | 1.14(1.07,1.22) |
|  | Non-linearity test  (p-value) ^§^ | | <0.001 | 0.002 | 0.001 | 0.001 |
|  | Chi2 for z-scores^#^ | | 80.76 | 73.13 | 57.06 | 56.19 |
|  | Chi2 for z-scores across quintiles^#^ | | 75.72 | 69.52 | 53.24 | 36.47 |
| Fatal CVD cases | | | | | | |
| Dietary pattern 1 | Linear | 838 | 1.11(1.05,1.16) | 1.10(1.05,1.16) | 1.07(1.02,1.13) | 1.07(1.02,1.13) |
|  | Q1 | 143 | 1.00(0.85,1.18) | 1.00(0.85,1.18) | 1.00(0.84,1.18) | 1.00(0.84,1.18) |
|  | Q2 | 161 | 1.11(0.95,1.29) | 1.11(0.95,1.30) | 1.09(0.93,1.27) | 1.09(0.93,1.27) |
|  | Q3 | 155 | 1.08(0.92,1.27) | 1.08(0.92,1.27) | 1.04(0.89,1.22) | 1.04(0.89,1.22) |
|  | Q4 | 179 | 1.29(1.12,1.50) | 1.29(1.11,1.49) | 1.21(1.04,1.40) | 1.21(1.04,1.39) |
|  | Q5 | 200 | 1.48(1.28,1.71) | 1.45(1.25,1.67) | 1.29(1.11,1.50) | 1.29(1.11,1.51) |
|  | Non-linearity test  (p-value) ^§^ | | <0.001 | 0.001 | 0.033 | 0.001 |
| Dietary pattern 2 | Linear | 838 | 1.06(1.00,1.12) | 1.06(0.99,1.12) | 1.06(1.00,1.13) | 1.06(1.00,1.13) |
|  | Quadratic | 838 | 1.02(1.01,1.04) | 1.02(1.00,1.04) | 1.02(1.01,1.04) | 1.02(1.01,1.04) |
|  | Q1 | 173 | 1.00(0.86,1.16) | 1.00(0.86,1.17) | 1.00(0.86,1.17) | 1.00(0.86,1.17) |
|  | Q2 | 154 | 1.00(0.86,1.17) | 1.01(0.86,1.18) | 1.01(0.86,1.18) | 1.01(0.86,1.18) |
|  | Q3 | 152 | 0.96(0.82,1.13) | 0.97(0.83,1.14) | 0.98(0.83,1.15) | 0.98(0.83,1.15) |
|  | Q4 | 169 | 1.03(0.89,1.20) | 1.03(0.89,1.20) | 1.06(0.91,1.23) | 1.05(0.90,1.22) |
|  | Q5 | 190 | 1.15(1.00,1.33) | 1.15(1.00,1.33) | 1.18(1.02,1.36) | 1.18(1.02,1.36) |
|  | Non-linearity test  (p-value) ^§^ | | 0.027 | 0.043 | 0.026 | 0.031 |
|  | Chi2 for z-scores^#^ | | 26.72 | 23.70 | 17.44 | 17.15 |
|  | Chi2 for z-scores across quintiles^#^ | | 18.79 | 16.70 | 10.48 | 6.56 |
| All-cause mortality cases | | | | | | |
| Dietary pattern 1 | Linear | 3629 | 1.11(1.09,1.14) | 1.11(1.08,1.14) | 1.08(1.05,1.10) | 1.08(1.05,1.11) |
|  | Q1 | 660 | 1.00(0.92,1.08) | 1.00(0.92,1.08) | 1.00(0.92,1.08) | 1.00(0.92,1.08) |
|  | Q2 | 703 | 1.08(1.00,1.16) | 1.08(1.00,1.16) | 1.06(0.98,1.14) | 1.06(0.98,1.14) |
|  | Q3 | 711 | 1.14(1.06,1.23) | 1.14(1.06,1.23) | 1.10(1.03,1.19) | 1.10(1.03,1.19) |
|  | Q4 | 740 | 1.27(1.18,1.37) | 1.26(1.18,1.36) | 1.19(1.11,1.28) | 1.19(1.11,1.28) |
|  | Q5 | 815 | 1.53(1.43,1.65) | 1.50(1.40,1.62) | 1.35(1.25,1.45) | 1.37(1.27,1.47) |
|  | Non-linearity test  (p-value) ^§^ | | 0.015 | <0.001 | 0.003 | 0.001 |
| Dietary pattern 2 | Linear | 3629 | 1.05(1.01,1.08) | 1.05(1.01,1.08) | 1.05(1.02,1.09) | 1.05(1.02,1.08) |
|  | Quadratic | 3629 | 1.01(1.00,1.02) | 1.01(1.00,1.02) | 1.01(1.00,1.02) | 1.01(1.00,1.03) |
|  | Q1 | 745 | 1.00(0.93,1.08) | 1.00(0.93,1.08) | 1.00(0.93,1.08) | 1.00(0.93,1.08) |
|  | Q2 | 671 | 0.98(0.91,1.06) | 0.98(0.91,1.06) | 0.99(0.92,1.07) | 0.98(0.91,1.06) |
|  | Q3 | 702 | 1.01(0.94,1.09) | 1.02(0.95,1.10) | 1.03(0.96,1.11) | 1.02(0.95,1.10) |
|  | Q4 | 767 | 1.09(1.02,1.17) | 1.09(1.02,1.18) | 1.12(1.04,1.20) | 1.11(1.03,1.19) |
|  | Q5 | 744 | 1.09(1.01,1.17) | 1.09(1.01,1.17) | 1.12(1.04,1.20) | 1.11(1.03,1.19) |
|  | Non-linearity test  (p-value) ^§^ | | 0.072 | 0.124 | 0.086 | 0.050 |
|  | Chi2 for z-scores^#^ | | 88.77 | 79.84 | 49.66 | 51.61 |
|  | Chi2 for z-scores across quintiles^#^ | | 78.22 | 70.39 | 41.46 | 26.01 |
| Note: Adjusted hazard ratios (HR) and 95% confidence intervals (CI) of total DP scores obtained using Cox proportional hazard regression. Adjusted HRs (hazard ratio) and confidence intervals (CI) of DP scores quintiles obtained using the floated absolute risk method of Cox proportional hazards regression. Age at risk adjusted for by using age during study as the underlying timescale for Cox regression. All the models were stratified by sex and regions (England, Scotland and Wales). *Sociodemographic characteristics: Ethnicity, Townsend index of deprivation, education. ^†^ Behavioral risk factors: smoking status, physical activity. ^‡^Full model was stratified by sex and regions (England, Scotland and Wales) and adjusted for ethnicity, socioeconomic status, behavioral risk factors, energy intake and menopause in women. ^§^Likelihood ratio test was used to test for a potential non-linear association between dietary patterns and health outcomes. ^#^ Chi2⁠ was calculated by likelihood ratio test, to measure the extent to which dietary pattern 1 and dietary pattern 2 predict CVD risk and all-cause mortality risk in different models (e.g. with and without adjustment for which dietary pattern 1 and dietary pattern 2) | | | | | | |

| 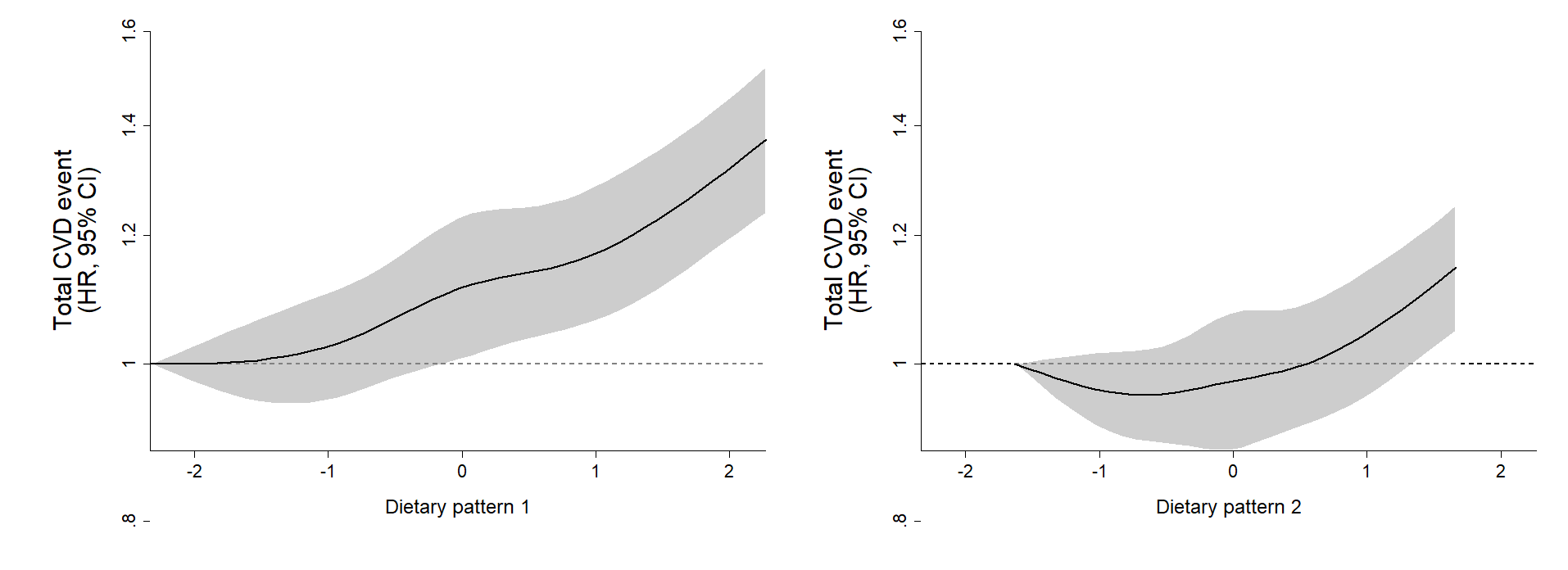 |
| --- |
| 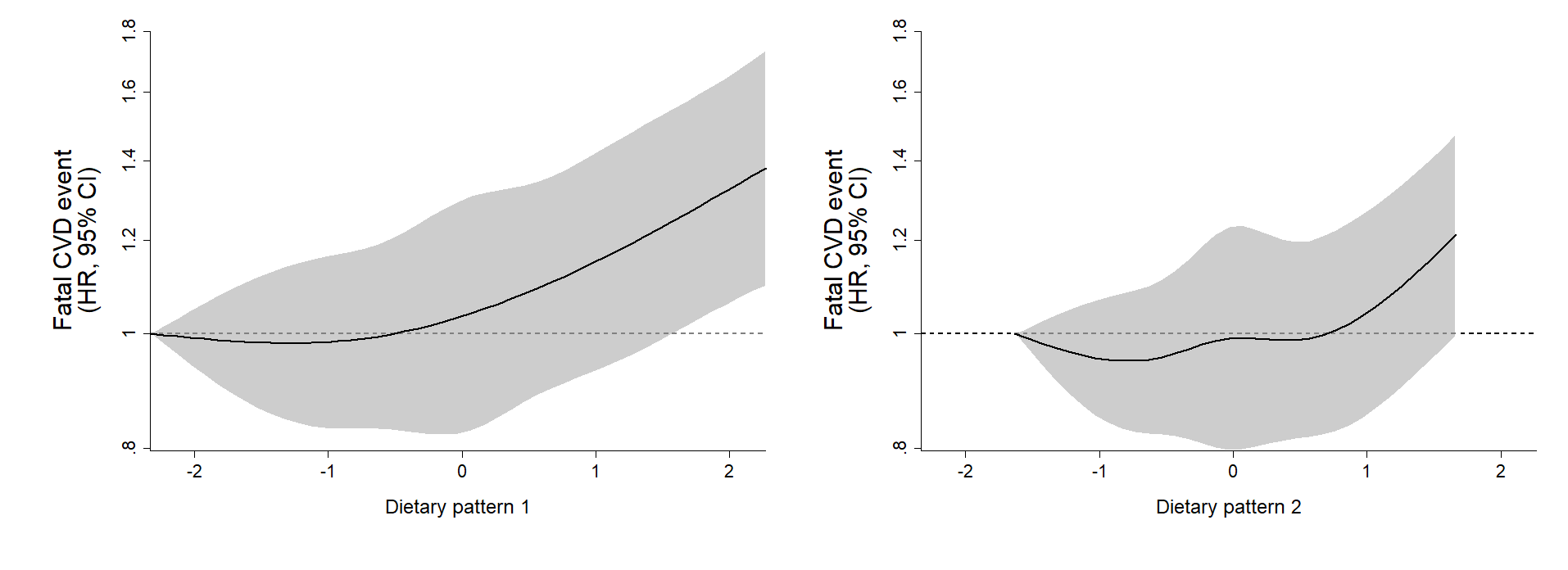 |
| 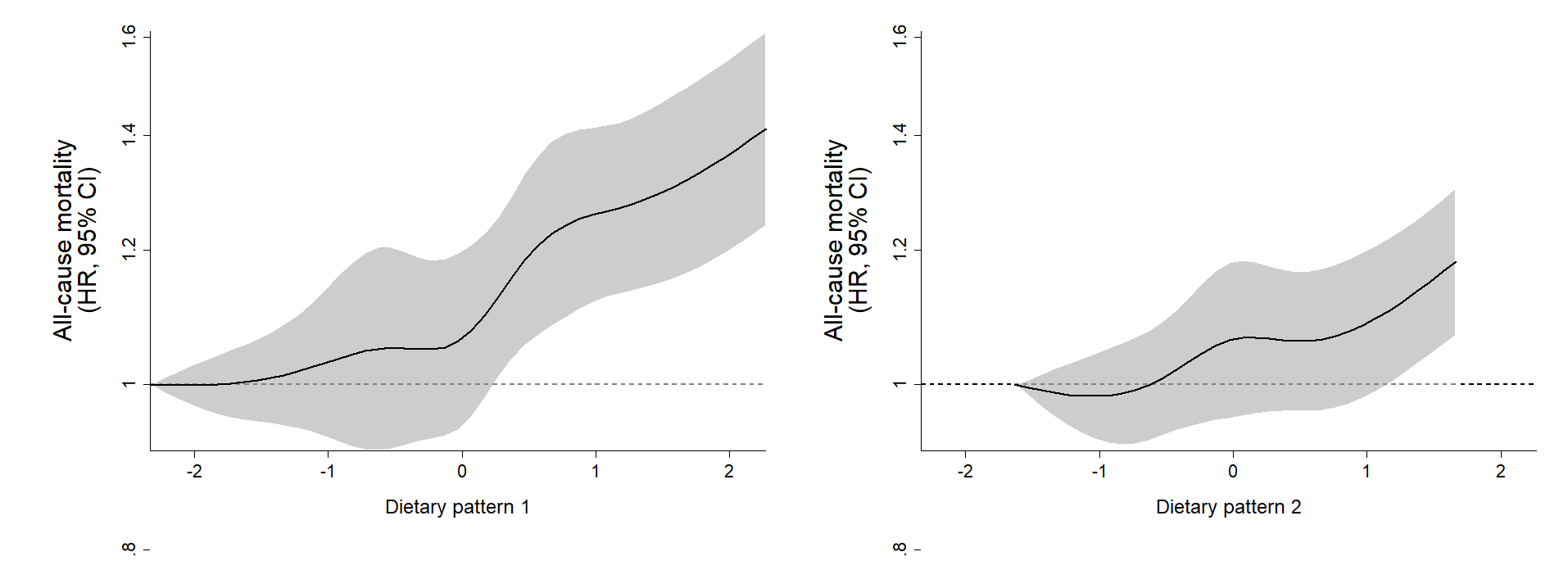 |
| **Additional Figure S6: HRs (95% CIs) of continuous dietary pattern scores for the risk of total and fatal CVD events and all-cause mortality (n=116 806).**  HRs (solid black lines) and 95% CIs (grey areas) were derived from spline regression models to examine the possible nonlinear relation of DP scores to total and fatal CVD events and all-cause mortality (stratified by sex and regions (England, Scotland and Wales) and adjusted for ethnicity, socioeconomic status, behavioral risk factors, energy intake and menopause in women). For simplicity of presentation, the reference values of DP scores were set to the 5% quantile of dietary pattern scores. Z scores for DP1 and DP2 were analyzed in mutually adjusted models to examine their independent associations with health outcomes. |

| Additional Table S7: Associations between dietary pattern scores and the risk of total and fatal CVD and all-cause mortality excluding people who had the event within two years after completing their last 24-h online dietary assessment (N=115 532) | | | | | | | | | |
| --- | --- | --- | --- | --- | --- | --- | --- | --- | --- |
|  | **Total CVD** | | | **Fatal CVD** | | | **All-cause mortality** | | |
|  | HR | 95% CI | | HR | 95% CI | | HR | 95% CI | |
| *Dietary pattern 1* |  |  |  |  |  |  |  |  |  |
| Linear | 1.06 | 1.03 | 1.09 | 1.06 | 1.00 | 1.12 | 1.08 | 1.05 | 1.11 |
| *Dietary pattern 2* |  |  |  |  |  |  |  |  |  |
| Linear | 1.04 | 1.00 | 1.07 | 1.05 | 0.98 | 1.12 | 1.05 | 1.02 | 1.08 |
| Quadratic | 1.02 | 1.01 | 1.03 | 1.03 | 1.01 | 1.04 | 1.01 | 1.00 | 1.03 |
| N of event | 2971 | | | 698 | | | 3407 | | |
| Notes:  All the models were stratified by sex and regions (England, Scotland and Wales) and adjusted for ethnicity, socioeconomic status, behavioral risk factors, energy intake and menopause in women. Z scores for DP1 and DP2 were analyzed in mutually adjusted models to examine their independent associations with health outcomes. | | | | | | | | | |

**
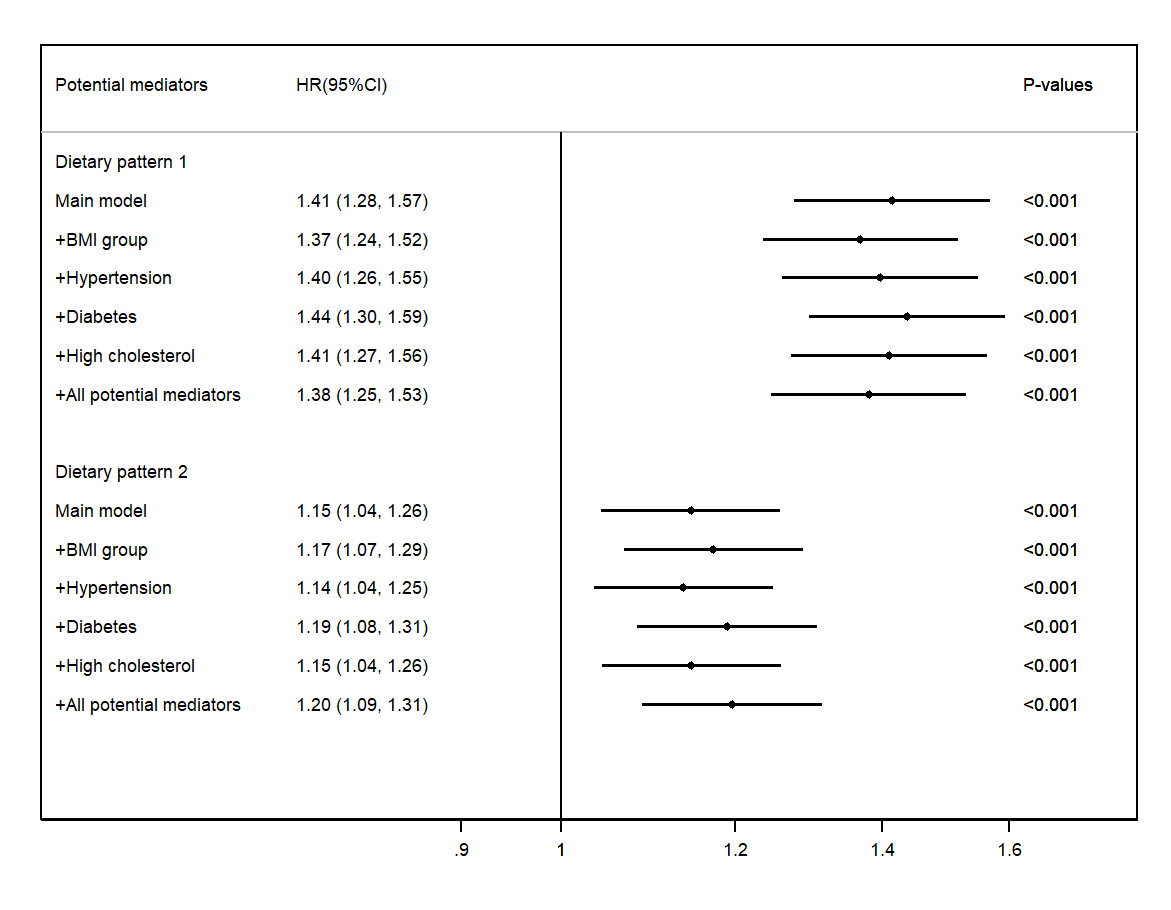
**

**Additional Figure S7 Relationships between dietary pattern scores in quintile 5 compared to quintile 1 and the risk of total CVD events, further adjusted for potential mediators of the associations**

All the models were stratified by sex and regions (England, Scotland and Wales) and adjusted for ethnicity, socioeconomic status, behavioral risk factors, energy intake and menopause in women. All potential mediators included BMI group, hypertension, diabetes, and high cholesterol. Dietary pattern 1 and dietary pattern 2 were analyzed in mutually adjusted models to examine their independent associations with health outcomes. P-values were calculated by likelihood ratio test.

**
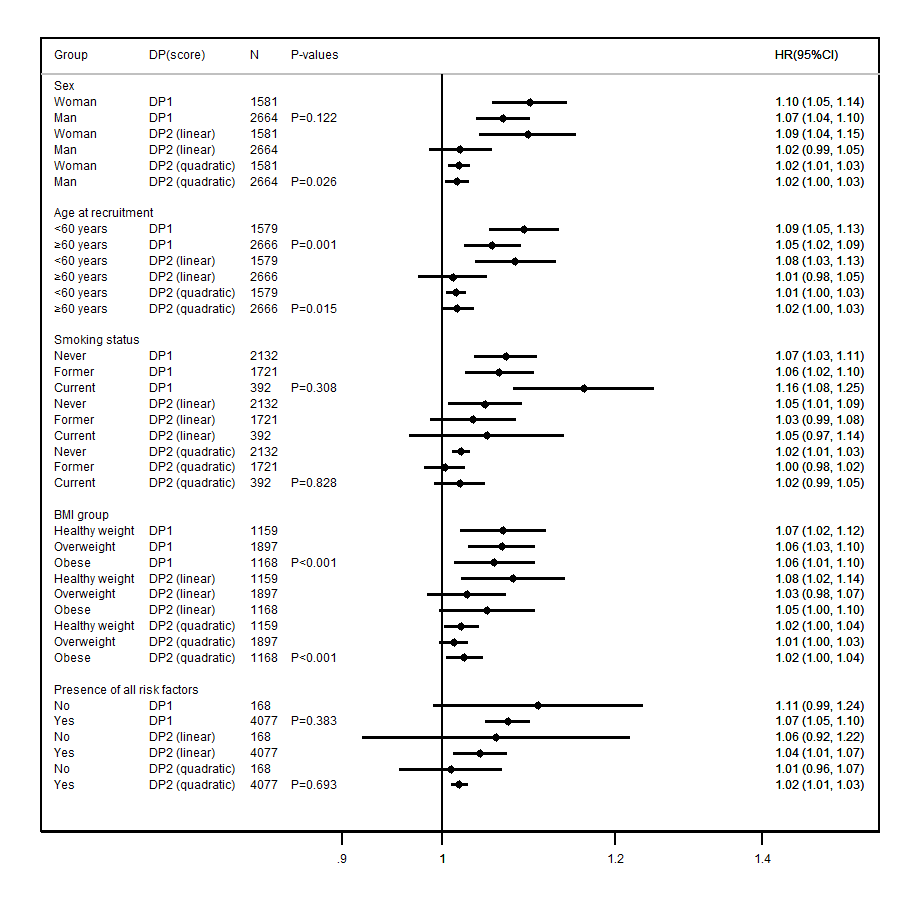
**

**Additional Figure S8: The association between dietary patterns and risk of total CVD by subgroups (N=116 806)**

All the models were stratified by sex and regions (England, Scotland and Wales) and adjusted for ethnicity, socioeconomic status, behavioral risk factors, energy intake, and menopause in women. Test of interaction by sex, age at recruitment, smoking, BMI group, presence of all risk factors (hypertension, diabetes and cholesterol lowering medication) were performed by adding appropriate interaction terms to the Cox models and testing for statistical significance of interaction across strata using likelihood ratio tests. Z scores for DP1 and DP2 were analyzed in mutually adjusted models to examine their independent associations with outcome. P-values were corrected by Bonferroni multiple test. HR value in underweight group was not presented due to few cases (N=21).
